# An integrative machine learning, explainable AI, molecular simulation, and cytotoxicity validation framework for the discovery of selective SIRT1 inhibitors against triple negative breast cancer

**DOI:** 10.3389/fbinf.2026.1827014

**Published:** 2026-06-10

**Authors:** Deepak Sharma, Madhan Subramaniam, Madhu Anabala, Rajiniraja Muniyan

**Affiliations:** 1 School of Bio-Sciences and Technology, Vellore Institute of Technology, Vellore, India; 2 Department of Computer Science and Engineering, University College of Engineering Thirukkuvalai, (A Constituent College of Anna University) Thirukkuvalai, Nagapattinam, India

**Keywords:** AI, cytotoxicity, machine learning, molecular docking, molecular dynamics simulation, SIRTUIN, TNBC

## Abstract

**Background:**

Triple negative breast cancer (TNBC) is an aggressive disease characterized by a poor prognosis, high decline rates, lack of hormonal receptors for targeted therapy, limited effectiveness of existing treatments, and the emergence of chemoresistance. Sirtuin1 (SIRT1) is an epigenetic modifier and nicotinamide adenine dinucleotide (NAD+) dependent class III histone deacetylase (HDACs) protein. It promotes the modulation of several tumour suppressors and oncogenes. The evidence also suggests that inhibiting SIRT1 activity using selective SIRT1 inhibitors could restore E-cadherin expression and suppress EMT-mediated metastasis in TNBC cells. Though we have many SIRT1 protein inhibitors, they exhibited off-target effects, low isoform selectivity, and inefficiency at the clinical trial stage.

**Methods:**

In this study, we aim to identify SIRT1 isoform inhibitors by utilizing an integrated computational approach. Three stages of ML modelling were performed to find the best model from the SIRT1-based dataset. The QDA + ROS and XGBClassifier + ROS models were identified as the most robust, and they were subjected to the SHAP framework (XAI approach) to address the “black box” nature of the developed ML models. The NPASS natural compound dataset was first screened with the applicability domain of the developed models, followed by a two-step virtual screening with UniDock and AutoDock GPU in Scientiflow. Finally, the selected compounds were taken for molecular dynamics simulation, with rigorous trajectory analysis, and preliminary experimental validation was done.

**Results:**

The compounds with NPASS IDs: NPC216682, NPC480509, NPC210910, and NPC247082 were identified as the most promising hits. Among these hits, Praziquantel (NPC480509), the only available test compound with reported anticancer properties, revealed the cytotoxic nature on MDA-MD-231 and MCF7 breast cancer cell lines, whereas non-cytotoxic on normal breast cell line (MCF10A).

**Conclusion:**

This study is exploratory. Exact SIRT1 selective inhibition by Praziquantel, along with other hits, may be further studies thorugh *in-vitro* and *in vivo* evaluation to understand the exact mechanism of action of these hits. The integrated *in silico,* and preliminary *in-vitro* approach proposed in this study could lead to innovative outcomes when applied in a pharmaceutical framework distinct from traditional methods.

## Graphical Abstract

**Figure:**
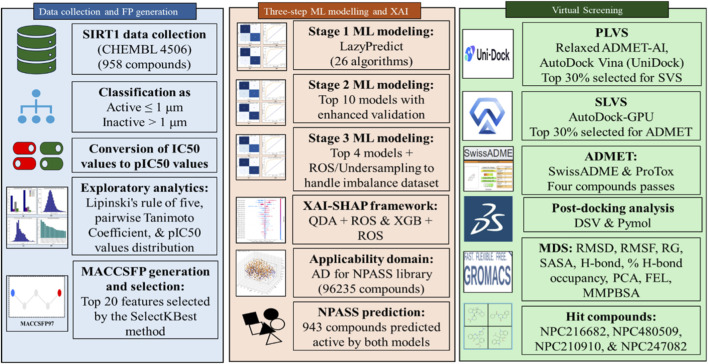

## 1 Background

As per the statistics released by the Global Cancer Observatory (GLOBOCAN) in 2022, breast cancer (BC) is the most common site of cancer worldwide, including India. In fact, BC exhibits nearly 27% of all cancers, which marks it as one of the most challenging maladies (Bray et al., 2024). The proportion of the most aggressive subtype of BC, i.e., Triple Negative Breast cancer (TNBC), which constitutes the absence of hormone receptors, such as Estrogen Receptor (ER), Progesterone Receptor (PR), and Human Epidermal Growth Factor Receptor 2 (HER2), ranges from 6.7% to 27.9% worldwide (Sandhu et al., 2016; Kulkarni et al., 2020). The highest proportions have been reported in India as well, underscoring the alarming nature of TNBC. The major risk factors for TNBC progression in the Indian population include modern lifestyles, obesity, substance abuse, family history, etc. (Thakur et al., 2018). The present treatment to combat TNBC includes conventional therapeutic approaches like cytotoxic chemotherapy, but the lack of hormone and HER2 targets makes these options unsatisfactory. High recurrence rates, off-target effects, and therapeutic resistance are other constraints to tackle TNBC (Yin et al., 2020).

The epigenetic modifications, such as DNA methylation, histone modification, and chromatin remodeling, play a crucial role in cell cycle progression, proliferation, apoptosis, and cell death. Furthermore, epigenetic modifications are the key hallmark in cancer therapy (Kala et al., 2015). A well-known epigenetic modifier is the Sirtuin (SIRT) proteins. SIRTs are the nicotinamide adenine dinucleotide (NAD^+^) dependent class III histone deacetylase proteins, which constitute a total of seven members (SIRT1 to SIRT7). SIRT family proteins can be classified by cellular location. For example, SIRT1, SIRT6, and SIRT7 are predominantly found in the nucleus, SIRT2 in the cytoplasm, and SIRT4 and SIRT5 in mitochondria. A fraction of SIRT1 may also be detected in the cytoplasm, and SIRT2 in the nucleus, under certain conditions of cell types and cell cycle stages (Dang, 2014; Wu et al., 2022). The SIRT protein inactivates target proteins, such as p53, through the deacetylation process in the presence of NAD+ cofactor. The deacetylated target protein, 2′-O-acetyl-ADP-ribose (2, O-acetyl-ADPR), and NAM result from the action of SIRT protein. SIRTs also regulate many other oncogenes and tumor suppressors, including “FoxO family, HES1, HEY2, PPARγ, CTIP2, p300, PGC-1α, and NF-κB,” in addition to p53 (Rahman and Islam, 2011). This made the SIRT protein a key target to regulate the functioning of oncogenes and tumor suppressors, and ultimately, cancer.

Among all the SIRTs, SIRT1 has been found to play a major role in BC regulation. SIRT1’s function in cancer biology is debatable. For example, malignancies of the breast, prostate, colon, and liver often express more SIRT1. In these tumors, SIRT1 plays a major part in encouraging the survival, growth, and invasion of cancer cells (Zhao et al., 2006). In contrast, a few reports also suggest the reduced expression of SIRT1 protein in other types of cancers. Another study reported that inhibiting SIRT1 also causes the E-cadherin gene to re-express, which further interacts with β-catenin in breast cancer and colon cancer cell lines. This interaction prevents WNT signaling from being activated further (Pruitt et al., 2005). Apparently, Sirtinol (a SIRT1 inhibitor) was found to be highly cytotoxic to 4T1 BC cells. It also reduced splenic metastatic growth by significantly decreasing vimentin expression and reducing the ability of 4T1 cells to migrate from the tumor site (Satam et al., 2024). Another study used a selective SIRT1 inhibitor, i.e., EX-527, which inhibited MDA-MB-231 cell proliferation and induced apoptosis. Results of immunoblotting revealed that SIRT1 expression was decreased, while p53 was increased, and POLD1 was decreased in the cells treated with EX-527 in the same study (Salih et al., 2024).

The possible reason for the controversial role of the SIRT1 protein could be “use of various cell lines, a group of human or animal samples with distinct genetic subtypes, tumor grades, and stages.” Overall, these studies suggested that inhibition of the SIRT1 protein could be a possible solution to manage TNBC. To date, a number of studies have been done to understand the function of SIRT1 in TNBC, but some research gaps need to be filled, such as a lack of SIRT-isoform-selectivity and specificity, restrained potency, limited bioavailability, poor pharmacokinetics and pharmacodynamics properties, and insufficient clinical and preclinical trials (Curry et al., 2021; Sharma et al., 2025b). This study aims to identify a SIRT1-isoform selective inhibitor using rigorous computational techniques.

Natural products are more appealing targets for therapeutic development because of their greater chemical diversity, biological specialization, and structural characteristics (Karunakaran and Muniyan, 2023a). A reported study by Sinha et al. focused on the plant-derived inhibitors such as apigenin, kaempferol, and sulforaphane and demonstrated a strong inhibitory effect against SIRT1, SIRT3, SIRT5, and SIRT6 through *in silico* and *in-vitro* approaches. These natural compounds possessed significant SIRT protein inhibition and ultimately decreased cellular viability, global activity, and protein expression of SIRTs and stemness of TNBC cells (Sinha et al., 2019). In another study, the Sulforaphane-Cisplatin (SFN-CIS) combination synergistically hinders cellular growth of TNBC cells, such as MDA-MB-231 and MDA- MB-468 cells (Sinha et al., 2021). Similarly, there are numerous studies which suggested the major role of natural compounds in inhibiting SIRT1 protein and, therefore, TNBC management.

Molecular docking, molecular dynamics simulations (MDS), artificial intelligence (AI), and machine learning (ML) are some of the computational methods used in the recently developed field of computer-aided drug design (CADD) (Vemula et al., 2023; Jain et al., 2025). Despite the advancement in SIRT1 biology in coordination with BC or TNBC, there are limited studies that deal extensively with advanced *in silico* techniques such as AI and ML. With these needs, our study aims to identify natural SIRT1 inhibitors using a three-stage ML modelling, explanation of the developed models using SHapley Additive exPlanations (SHAP) framework [explainable AI (XAI)], applicability domain of the unknown dataset, and prediction as active or inactive using developed SIRT1-based models as ligand-based drug design (LBDD). The predicted active compounds were investigated for structure-based drug design (SBDD), such as primary-level virtual screening, secondary-level virtual screening, pharmacokinetic and pharmacodynamic predictions, and Molecular Dynamics Simulation (MDS). The MDS studies were followed by several trajectory parameter analyses and binding free energy calculations to reveal four Hit compounds as the outcome of this study. Among these hits, one of the available hits (Praziquantel) was tested for preliminary experimental validation by cell viability and toxicity assay. Overall, this study provides a preliminary road map for validating identified active hits for SIRT1-based inhibitory activity through *in-vitro* and *in vivo* approaches.

## 2 Materials and methods

### 2.1 Raw data collection and curation

To perform the ML-based QSAR model for SIRT1 protein, we have retrieved only bioactivity data for *Human SIRT1* (UniProt ID: Q96EB6) (ChEMBL ID: CHEMBL4506). The ChEMBL web source client was utilized to execute this step (Gaulton et al., 2012). We gathered a total of 958 compounds that were experimentally proven to modulate SIRT1 biological activity. The dataset included IC_50_ values, which are considered the gold standard for assessing the inhibitory activity of any protein. The IC_50_ value was mentioned in nanomolar (nm) for the entire dataset.

The provided dataset could not be used as is because it requires preprocessing steps, such as removing inorganic salts, mixtures, and organometallics. This step ensured the acceptability of the input dataset by widely used descriptor-generating tools. The focus was also on removing duplicate compounds, extreme values, and missing values through manual inspection. As per the published protocols, we assigned all 958 compounds as active or inactive. For this, the bioactivity data’s IC_50_ in nm were converted to the IC_50_ value in μM. Then, the compounds with IC_50_ higher than 1 μM were considered inactive, and the ones with IC_50_ below or equal to 1 μM were considered active (Machado et al., 2022). With this, out of 958 compounds, we had 155 compounds as active and 803 compounds as inactive. Furthermore, IC_50_ values were converted to negative logarithmic units (pIC_50_ = −logIC_50_ × 10^–9^), which made the dataset more evenly distributed. These steps were executed in slightly modified Python scripts.

### 2.2 Exploratory analytics

The dataset was further evaluated based on the drug likeness analysis using Lipinski’s rule of five (RO5), pairwise Tanimoto Coefficient (TC), and pIC_50_ values distribution profiles. Firstly, the molecular descriptors [Molecular weight (MW), LogP, Hydrogen bond donors (HBD), and Hydrogen bond acceptors (HBA)] were calculated for the entire dataset using the RDKit package. The graph was plotted for active and inactive compounds that exhibited 0 to 5 RO5 violations. A comparison of RO5 for the active and inactive datasets was considered to confirm the reliability of the input dataset. The TC is a similarity metric with values ranging from 0 (no similarity) to 1 (same) that measures the overlap between two sets as the ratio of their shared characteristics to the total unique features. We calculated the Tanimoto Coefficient for the entire dataset to confirm the distribution of the input dataset based on TC values. Lastly, a plot between pIC_50_ values and frequency was also made to understand the distribution of the input dataset based on pIC_50_ values. These steps were executed with partially modified Python scripts as provided in our GitHub repository.

### 2.3 Feature generation and feature selection

A molecular descriptor is a numerical representation of a specific physicochemical, structural, or topological property of a particular chemical moiety. Apparently, a molecular fingerprint (FP) is a binary representation that shows either the presence or absence of a specific structural feature or pattern in a molecule. Descriptors are more suited for regression and physicochemical property prediction, whereas fingerprints are best for similarity-based screening and classification (Cereto-Massagué et al., 2015). In the current study, we generated the Molecular ACCess System fingerprint (MACCSFP). The circular fingerprints (e.g., Morgan) and physiochemical descriptors offer broad coverage, whereas the MACCSFP were chosen due to their structural interpretability (Cereto-Massagué et al., 2015). We used PaDEL-Descriptor (padelpy-0.1.16) with the Chemistry Development Kit (CDK) to generate MACCSFP for all 958 reported SIRT1-based compounds (Yap, 2011).

The FP generation was followed by a feature selection step, which ensured the greater statistical significance of the developed ML model. This step also helps to reduce the computational cost and the overfitting of the trained models. To get rid of the model complexity, we removed low-variance features (threshold >0.10) and intercorrelated features (>0.9) from our dataset using the scikit-learn (sklearn) library in Python (Pedregosa et al., 2011; Karunakaran and Muniyan, 2023b). Furthermore, a Mutual info classifier was used to further narrow down the most important feature. The scoring function, SelectKBest, was applied to identify the top 20 MACCSFP for the SIRT1-based dataset. These top 20 features were further used throughout the study. These top 20 features contain the optimum number of features, which were later confirmed by the robustness of the built models.

### 2.4 Model development

The entire dataset filtered with the top 20 MACCSFP was loaded in Google Colab (https://colab.research.google.com/) for further execution of Python scripts. The dataset was divided into a train and a test set in the ratio 70:30 using a random seed of 42, which gave reproducible splitting of the dataset. After splitting the data, we had a total of 670 compounds in the train and 288 compounds in the test set. A three-dimensional Principal Component Analysis (3D-PCA) plot based on the MACCS feature for the test and the train set was generated to understand the diversity of the chemical space in the train and the test set. Furthermore, the test set was used to validate the built model based on the train set in subsequent steps. The stage 1 modeling was performed using LazyClassifier in LazyPredict (Shankar Rao Pandala, 2021). The lazy classifier takes less time and is more accurate. There are 32 algorithms in the lazy classifier, of which 26 were used for the initial development based on data suitability. The LazzyClassifer also enabled us to identify the most promising algorithm for further investigations. The developed models were cross-validated using the test set. The validation parameters, such as accuracy, balanced accuracy, Receiver Operating Characteristic-Area Under the Curve (ROC-AUC), F1 score, and time taken, were considered for model evaluation. After the validation step, the top 10 models developed in the LazyClassifier steps were selected for further evaluations. Stage 2 modeling was executed for individual models, with their respective algorithms. The models were re-trained with enhanced validation metrics, including precision, recall, F1 score for active and inactive predictions, support, Cohen’s Kappa, Mean Absolute Error (MAE), and ROC-AUC curve.

Unfortunately, the stage 2 modeling did not lead to fruitful outcomes. The developed model so far exhibited good accuracy, precision, and ROC-AUC, but precision, recall, and F1 values were significantly varying for active and inactive models. The developed models were biased in stage 2 ML modeling. This was due to an imbalanced SIRT1-based data set (Guan and Fu, 2022; Gomatam et al., 2024). The models learned the pattern of inactive molecules more precisely than the active molecules. To overcome these limitations, we incorporated two sampling strategies (Random Over Sampler (ROS) and Random under-sampling) in our models as suggested in the recent studies (Machado et al., 2022; Sharma et al., 2025a). ROS balances an imbalanced dataset by duplicating samples from the minority class, increasing its representation, whereas under-sampling balances the data by removing samples from the majority class, reducing its dominance. This way, we could ensure a more robust SIRT1-based ML model for further virtual screening steps.

Therefore, another stage of ML modelling, i.e., stage 3, was added, which constituted the top 4 models (Random Forest Classifier, Bagging Classifier, Quadratic Discriminant Analysis (QDA), XGB Classifier: selected based on the overall evaluation parameters in stage 2) in combination with ROS and undersampling sampling strategies. The sampling was done specifically for the train set to avoid data leakage. The developed models were re-evaluated for all the validation parameters as in the previous stage. This third layer of ML modelling helped us to deal with biased models and an imbalanced dataset. An additional ten-fold cross-validation of all models built in stage 3 was done using the Stratified k-Fold method. To further confirm the statistical significance and to reduce the chances of correlation of the developed ML models in stage 3, Y-randomization (permutation testing) was also performed. Here, the bioactive labels (active or inactive) were randomly shuffled while the independent variables (MACCSFP) were made constant. This thing was repeated for 50 iterations. Finally, the robustness of the models was defined by comparing baseline (real labels) accuracy and average accuracy on randomized labels. If the baseline accuracy is greater than the average accuracy on randomized labels, then the model has robustness against chance correlation. The top 2 robust models that passed all the validation parameters were saved using the pickle module and evaluated with recently used Explainable-Artificial Intelligence (XAI).

### 2.5 Explanation of the developed models using the XAI method

The model QDA +ROS and XGBClassifier + ROS performed well and passed all the validation parameters for SIRT1-based ML models. These models were further explained using XAI based on SHapley Additive exPlanations (SHAP) framework, and therefore, they addressed the “black box” character of the SIRT1-based ML models. This framework gives weight to each feature used in building a model, based on how important it is to the model’s prediction. SHAP values provide information about the model’s decision-making process by showing the anticipated change in prediction when a certain feature is included vs. removed (Lundberg and Lee, 2017; Lundberg et al., 2020). The XAI approach helped us to explain the predictions made by SIRT1-based ML models.

### 2.6 Retrieval of unknown compounds

Natural Product Activity and Species Source (NPASS) is a freely accessible database that was first launched in the year 2018. After that, it was updated in the years 2023 and 2025 (Zhao et al., 2023). A total of 96,234 natural compounds were retrieved from the NPASS 2.0 database (retrieved date April, 2025), and the MACC fingerprints were calculated for all the compounds. The top 20 features, as previously mentioned, were selected for further predictions.

### 2.7 Applicability domain

To assess the applicability domain (AD) of the ML models, we employed a nearest-neighbor methodology based on Tanimoto similarity coupled with a PCA analysis of chemical space. The train set and NPASS natural compounds were used to extract MACCS fingerprints. The top 20 MACCS fingerprints that were present in both the training and NPASS databases were considered in order to ensure consistency in the features. The Tanimoto similarity, computed on a pairwise basis from the train set, was used to calculate the maximum similarity for each compound to its highest Tanimoto neighbor in the training set. The AD was set as the fifth percentile of the complete distribution of maximum Tanimoto similarities across the training set, thus characterizing the lowest structural similarity that is acceptable by the model.

Using the approach stated above, the maximum Tanimoto similarity of each NPASS molecule to all the molecules in the training set was determined. Molecules exhibiting Tanimoto similarity values greater than the AD threshold were subsequently classified as being within the AD (NPASS compound - IN), whereas those exhibiting lower Tanimoto similarities were classed as outside the AD (NPASS compound - OUT). To further support the structural similarity between the training and NPASS molecules visually, PCA was performed on the combined training and NPASS molecular fingerprint datasets, and the top three principal components were plotted in 3D space along with a bounding box to depict the central training area. By performing the analyses and structure-sharing evaluations described above, we ensure that only structurally similar unknown compounds were considered for the prediction step by the SIRT1-based ML model.

### 2.8 Prediction of NPASS database natural compounds

The natural compounds, which were identified as “NPASS compound - IN,” were classified as active (1) or inactive (0) using QDA + ROS and XGBClassifier + ROS ML models. The compounds were thoroughly evaluated for their potential to yield active predictions using the two models, and those identified as active by both models were subsequently considered in our study.

### 2.9 Primary level virtual screening using UniDock

The crystal structure of the SIRT1 protein was retrieved from the PDB database (https://www.rcsb.org/) with PDB ID: 4I5I in PDB format (Berman et al., 2000; Zhao et al., 2013). The obtained structure of the SIRT1 protein had a resolution of 2.50 Å and consisted of two similar chains, each with 287 amino acids. The structure also contained three small molecules: nicotinamide-adenine-dinucleotide (NAD), (6S)-2-chloro-5,6,7,8,9, 10-hexahydrocyclohepta[b]indole-6-carboxamide (indole analogue 35) (4I5), and zinc ion (Zn^2+^) for structural stability. The protein preparation was done in the AutoDock 4.2 tool (Morris et al., 2009). To do so, water molecules and heteroatoms were removed from the structure, whereas polar hydrogens and Kollman charges were added to chain A of the SIRT1 protein’s crystallographic structure. The structure was saved in PDBQT for further use. The SIRT1’s catalytic binding site and validation of the docking protocol have already been done in our previous study. The validation protocol using the co-crystallized ligand in the binding pocket of the SIRT1 protein provided the RMSD values of 2.497 Å. Generally, the RMSD value obtained after the superimposition of the crystallographic pose and the docked pose nearby 2 Å, is suggested to be significant. Therefore, the same dimensions were used in the current study as Center X × Center Y × Center Z (35.001 × −16.391 × 26.347), NPTS X × NPTS Y × NPTS Z (76 × 90 × 57), and grid spacing of 0.375 Å (Sharma and Muniyan, 2024).

The primary and secondary level of virtual screening was performed in ScientiFlow: A scientific workflow automation tool (available at: https://scientiflow.com). The ScientiFlow’s “Virtual Drug Screening Pipeline” is designed to streamline the hit identification process by integrating ADMET prediction, GPU-accelerated docking, AI-enhanced refinement, and protein-ligand interaction analysis into a single automated platform. We have used this tool to ensure efficiency, scalability, and reproducibility while handling tens of thousands of ligands in a short time frame. The input for this tool is a list of compounds in CSV format, which it will process in accordance with the specifications to provide the desired output. The initial screening started with AI-based ADMET filtering using the ADMET-AI tool (Swanson et al., 2024). To narrow down chemical space in a lenient way, relaxed constraints of Known-drug-like (MW ≤ 800, logP ≤ 6.5, HBA ≤ 7, HBD ≤ 15, TPSA < 180) were applied to the filtered natural compounds from the previous step. The filtered natural compounds after this stage were considered for the molecular docking-based primary level of virtual screening. The AutoDock Vina-based scoring function enabled with GPU and the number of modes ten were utilized in the primary level of screening using the Uni-Dock tool in the ScientiFlow platform (Yu et al., 2023). To further deepen insights, the top 30% of the compounds were taken for the next step of secondary-level virtual screening.

### 2.10 Secondary level virtual screening using AutoDock-GPU

The natural compounds obtained after the primary level virtual screening were considered for secondary level screening using AutoDock-GPU (Santos-Martins et al., 2021). AutoDock-GPU, in contrast to AutoDock 4.2, has a significant computational performance boost by utilizing the highly parallel design of Graphics Processing Units (GPUs). This enabled the large-scale virtual screenings at an accelerated rate, which are not feasible with the CPU-only version. However, both the software utilize Lamarckian Genetic Algorithm for performing protein-ligand based molecular docking. The secondary level virtual screening was executed with the same dimensions as in the previous step, and an exhaustiveness of 100 poses. Secondary-level virtual screening allowed us to analyze every protein-ligand complex in a detailed manner. After this step, the top 30% of the natural compounds were retained for further rigorous ADMET evaluations.

### 2.11 ADMET predictions

The shortlisted natural compounds were considered for ADME predictions using the SwissADME web tools (Daina et al., 2017). The compounds were filtered based on GI absorption, Lipinski, and Pan-assay interference compounds (PAINS) criteria in SwissADME. The compounds that crossed this stage were assessed for different toxicities, such as hepatotoxicity, carcinogenicity, immunotoxicity, mutagenicity, and cytotoxicity using the ProTox 3.0 webserver (Banerjee et al., 2024). The compounds that crossed the ADMET barrier were cross verified using interaction profiles.

### 2.12 Post-docking analysis

The top docking complexes were analyzed for types of interactions, number of bonds, size of the binding pocket, and superiority among the natural compounds compared with the reference compound. Software like Discovery Studio Visualizer (DSV) (https://discover.3ds.com/discovery-studio-visualizer-download) and Pymol (https://www.pymol.org/) were used to execute these interpretations.

### 2.13 Molecular dynamics simulations

The molecular docking tool considers the protein to be rigid and the ligand to be flexible, whereas in a real scenario, both are flexible in nature. Therefore, to mimic human physiology accurately, an extended 200 ns molecular dynamics simulation was performed for the selected protein-ligand complexes and SIRT1 in its apo-state. The GROMACS 24.3 version was utilized for this purpose (Abraham et al., 2015). The ligand topology was generated using the SwissParam web tool (Zoete et al., 2011), whereas the protein topology was generated using pdbtogmx GROMACS utility with CHARMM27 force field and TIP3P water molecules (Lindahl et al., 2010). The prepared protein-ligand complex was manually merged and kept in a cubic box of 1 nm. The protein-ligand complex was further neutralized with sodium (Na^+^) and chloride (Cl^−^) ions, followed by solvation and energy minimization for 50,000 steps. Furthermore, NVT and NPT equilibration steps were executed for 50,000 steps by applying position restraints. This step was executed at 300 K temperature and 1 bar reference pressure for 100 ps. Finally, the MDS production run command was run for 200 ns. After the completion of the MDS run, all the trajectories were analyzed for Root mean square deviation (RMSD) of backbone residues, Root mean square fluctuations (RMSF), Radius of gyration (Rg), Solvent accessible surface area (SASA), and the number of Hydrogen bonds were calculated and plotted using Xmgrace (https://plasma-gate.weizmann.ac.il/Grace/) and VMD software (Humphrey et al., 1996).

To capture the global motion of the C-alpha atoms of the SIRT1 protein in the apo state and halo state, Principal Component Analysis (PCA) was done. For this, a “gmx covar” and “gmx anaeig” modules were used along with the first ten PCs in each system. A plot of eigenvectors (direction of motion) and eigenvalues (amplitude of motion) was plotted after the generation of the covariance matrix. Apparently, Free Energy Landscape (FEL) plots were generated using “gmx sham” scripts in GROMACS. The FEL plots were visualized using Matlab software (https://in.mathworks.com/products/matlab.html) (Borkotoky and Murali, 2017; Singh et al., 2023).

### 2.14 MMPBSA calculation

The selected protein-ligand complexes were examined for the Molecular Mechanics Poisson-Boltzmann surface area (MMPBSA) analysis to calculate the total free binding energies using the gmx_MMPBSA module (Valdés-Tresanco et al., 2021). Using GROMACS-generated data, gmx_MMPBSA used MMPBSA.py by AMBER to estimate the total binding free energy (Miller et al., 2012). The MDS trajectory’s last 20 ns (2,000 frames) were used to compute the total binding free energy, which remained steady during the simulation.

### 2.15 MTT assay

The compound Praziquantel was procured from Tokyo Chemical Industry (TCI) (product number: P2125). The human breast cancer cell line (MDA-MB-231 and MCF7), and the human normal breast cell line (MCF10A) were obtained from the National Centre for Cell Science (NCCS), Pune, and grown in Eagle’s Minimum Essential Medium containing 10% fetal bovine serum (FBS). The cells were maintained at 37 °C, 5% CO_2_, 95% air, and 100% relative humidity. After 24 h, the cells were treated with serial concentrations (12.5 μg/mL to 200 μg/mL) of the test compound, and the plates were incubated under the same conditions. The medium containing no test compound served as a negative control, and all the tests were performed in triplicate. 3-[4,5-dimethylthiazol-2-yl]2,5-diphenyltetrazolium bromide (MTT) is a yellow tetrazolium salt that dissolves in water. Succinate-dehydrogenase, a mitochondrial enzyme found in living cells, cleaves the tetrazolium ring, turning MTT into an insoluble purple formazan. As a result, the quantity of formazan generated is directly correlated with the number of living cells. Following a 48-h incubation period, each well received 15 µL of MTT (5 mg/mL) in phosphate-buffered saline (PBS), which was then incubated for 4 h at 37 °C. After flicking off the MTT media, the formazan crystals were dissolved in 100 µL of DMSO, and the absorbance at 570 nm was measured using a microplate reader. The percentage cell viability and % cell inhibition were then calculated with respect to the control. A nonlinear regression graph was plotted between % Cell inhibition and Log concentration, and IC_50_ was determined using GraphPad Prism software (Mosmann, 1983; Monks et al., 1991).

## 3 Results

### 3.1 Data collection and exploratory analytics

**FIGURE 1:**
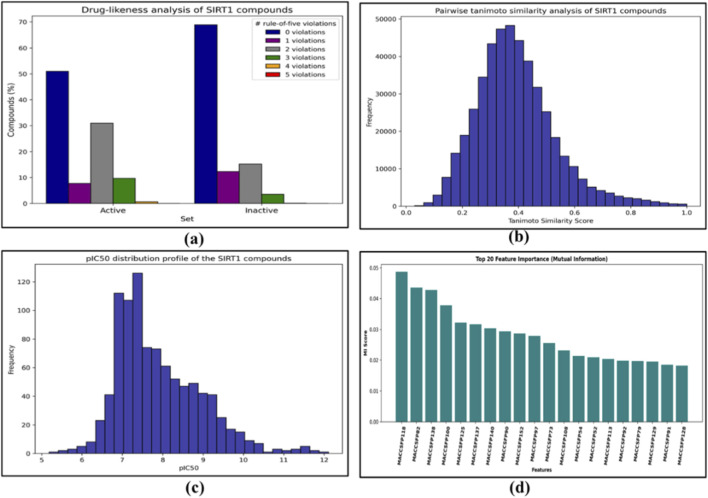
Exploratory analytics **(a)** drug-likeness analysis, **(b)** pairwise Tanimoto similarity analysis, **(c)** pIC50 distribution profile, and **(d)** top 20 features selected for the SIRT1-based chemical space.

We collected a total of 958 compounds that have shown potential effects on the SIRT1 protein using the ChEMBL web source client. The SIRT1-based compound library was categorized into 155 active compounds and 803 inactive compounds. The entire dataset was subjected to exploratory analytics. The dataset was analyzed for drug likeness, where we found that out of 155 active compounds, around 55% of the compounds showed zero violation of RO5, whereas 7.80%, 26.24%, and 9.92% of the active compounds exhibited 1, 2, and 3 violations of RO5. In the inactive dataset, around 69.57% of the inactive compounds showed zero RO5 violation, whereas 12.62%, 14.01%, and 3.66% of the inactive compounds showed 1, 2, or 3 RO5 violations (Figure 1a). Based on the drug likeness analysis of the active and inactive compounds, we could observe that most of the SIRT1-based input dataset of both active and inactive compounds lay in zero violation of RO5. This ensured that a reliable input dataset has been obtained, which might offer a promising orally administered drug for SIRT1 inhibition. Furthermore, pairwise TC similarity data were calculated and plotted against frequency for the entire SIRT1 compounds. In Figure 1b, it could be observed the uniform distribution of TC values for the entire dataset. The converted IC_50_ values to pIC_50_ values were further plotted against frequency. The pIC_50_ values distribution profile of SIRT1 compounds showed diversity in the pIC_50_ values in the input dataset (Figure 1c). Overall, the exploratory analytics confirmed the overall structure of the input dataset for building ML models.

### 3.2 Feature selection

Around 166 MACCSFPs were generated for each compound from the SIRT1-based input dataset. The feature generation step was followed by the feature selection step using the SelectKBest scoring function in Scikit-learn. The top 20 features were selected, which had the highest SelectKBest scores (Figure 1d; Supplementary Table S1). The SIRT1 input dataset with these top 20 Features was further used throughout the study.

### 3.3 Model development

**FIGURE 2:**
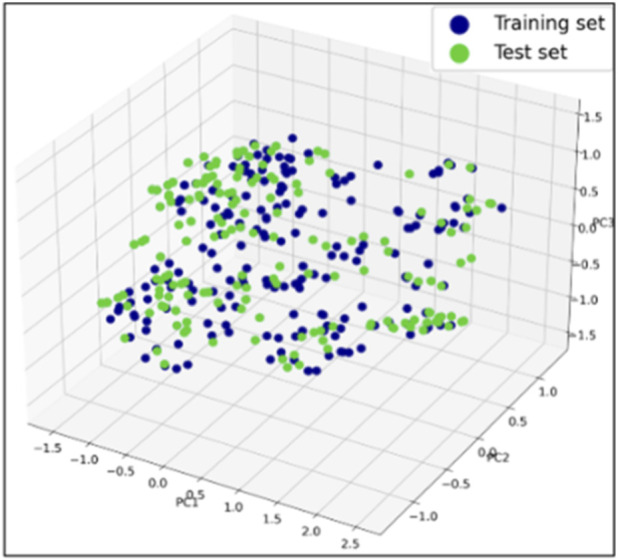
3D PCA analysis of train and test after splitting the data into a 70:30 ratio.

The model development step was done using all 958 SIRT1 compounds. The dataset was split into a train and a test set in a 70:30 ratio using a random seed of 42, resulting in repeatable dataset splitting. We had 288 compounds in the test set and 670 compounds in the train after splitting the data. The train and the test set were analysed in terms of diversity in the chemical space. The 3D PCA plot illustrated a high degree of overlap between train and test sets, i.e., with respect to the first three principal components (PCs). This suggested uniform chemical space coverage and model generizability without sampling bias and demonstrated the validity of the split method used for the dataset to be robust and therefore agreed with the assessment and outcome of the LazyClassifier application for evaluating the performance of subsequent ML models developed using this dataset (Figure 2).

#### 3.3.1 Stage 1 ML modelling

The train set was used for building the models, whereas the test set was utilized to validate the trained models in stage 1 ML modeling in LazyClassifier (LazyPredict). A total of 26 different algorithms were used in stage 1 ML modeling to build initial ML models. The developed models were validated with accuracy, balanced accuracy (BA), ROC-AUC, F1 score, and time taken. The detailed outcomes obtained from stage 1 ML modeling are provided in Supplementary Table S2. The top 10 models from stage 1 were selected for the stage 2 ML model with enhanced validation parameters to confirm the robustness of the built model.

#### 3.3.2 Stage 2 ML modelling

The top 10 selected models from stage 1 ML modelling were Label Spreading, Label Propagation, Random Forest Classifier, LGBM Classifier, Bagging Classifier, Bernoulli NB, Quadratic Discriminant Analysis, Decision Tree Classifier, Extra Trees Classifier, and XGB Classifier. The same SIRT1-based dataset was again used to rebuild the top 10 selected models in stage 2 ML modeling. The retrained models were validated with enhanced metrics, such as precision, recall, F1 score for active and inactive predictions, support, Cohen’s Kappa, Mean Absolute Error (MAE), and ROC-AUC curve. The performance of stage 2 ML modelling is mentioned in Supplementary Table S3. From the outcome, it could be seen that the top 10 models’ performance in terms of accuracy, BA, and ROC-AUC was closer to the stage 1 ML modelling. But, additional layers of validation, i.e., evaluating precision, recall, and F1, each with respect to active and inactive prediction, were not up to the mark. The precision, recall, and F1 values of the inactive compound prediction were near 0.90 for most of the models, whereas the precision, recall, and F1 values for the active compound prediction were rarely greater than 0.6. The ideal value of precision, recall, and F1 values for both active and inactive prediction should be closer to one. The developed models could predict inactive compounds more efficiently than active compounds. The possible reason for this was an imbalanced SIRT1-based dataset (Guan and Fu, 2022; Gomatam et al., 2024; Sharma et al., 2025a). Among the train and test sets, there were more inactive molecules than active molecules. The accuracy, BA, and ROC-AUC gave a significant outcome based on inactive compound predictions than on active compound predictions. Therefore, it gave similar results to those of stage 1 modelling. While the models did not perform well with precision, recall, and F1 values of the active compound prediction.

Despite this biased prediction, we could select the top 4 models, which outperformed ten models in stage 2 ML modelling, and these were Random Forest (RF) Classifier, Bagging Classifier, Quadratic Discriminant Analysis (QDA), and XGBClassifier (Supplementary Table S4). The overall performance of these four models in terms of accuracy, BA, ROC-AUC, Cohen’s Kappa, MAE, Precision (0), Precision (1), Recall (0), Recall (1), F1 (0), and F1 (1) was comparatively better. The further robustness of these top 4 models could be enhanced by handling the imbalanced input dataset in stage 3 ML modelling (Guan and Fu, 2022; Gomatam et al., 2024).

#### 3.3.3 Stage 3 ML modelling

As recommended by previous research, we included two sampling techniques (Random Over Sampler (ROS) and Random Under-sampling) in our models to overcome the restrictions in stage 2 modelling. While under-sampling balances the data by eliminating samples from the majority class, which lessens its dominance, ROS balances an imbalanced dataset by replicating samples from the minority class, boosting its representation. In this manner, we could guarantee a more reliable SIRT1-based machine learning model for subsequent virtual screening stages (Sharma et al., 2025a). The top four models [Random Forest Classifier, Bagging Classifier, Quadratic Discriminant Analysis (QDA), and XGB Classifier] were combined with ROS and undersampling techniques to create stage 3 of ML modeling. As in the previous step, the developed models were reassessed for every validation parameter. Among all eight combinations (Supplementary Table S4), QDA + ROS and XGBClassifier + ROS demonstrated the greatest robustness. The validation parameters, such as accuracy, BA, ROC-AUC, Cohen’s Kappa, and MAE, were found to be 0.8195, 0.8195, 0.9091, 0.6390, and 0.1804; and 0.8651, 0.8651, 0.9332, 0.7302, and 0.1348 for QDA + ROS and XGBClassifier + ROS ML models, respectively. More importantly, Precision (0), Precision (1), Recall (0), Recall (1), F1 (0), and F1 (1) values for QDA + ROS and XGBClassifier + ROS models were estimated as 0.79, 0.86, 0.88, 0.76, 0.83, and 0.81; and 0.85, 0.88, 0.88, 0.85, 0.87, and 0.86, respectively. Both QDA + ROS and XGBClassifier + ROS had equal potential to classify the compounds as active or inactive with equal capability. Therefore, this third layer of ML modelling helped us to deal with biased models and an imbalanced dataset.

An additional ten-fold cross-validation (CV) of all models built in stage 3 was done using the Stratified k-Fold method. The CV accuracies of QDA + ROS and XGBClassifier + ROS were identified as 0.7318 ± 0.0644 and 0.7273 ± 0.0686. Few models exhibited CV accuracies better than the above-mentioned models, but those were again biased to active and inactive prediction. The hold-out test set had higher predictive performance compared to the ten-fold cross-validation, but more variability among the folds (∼0.73 accuracy). Differences in the chemical distribution and representation of classes between the validation folds and the external hold-out set may be responsible for some of the variability. However, the models consistently showed predictive performance above other classification models across all folds, which indicated that even though there was moderate variability, the structure-activity relationships learned by the models were still meaningful.

Furthermore, Y-randomization was also performed to understand the robustness against chance correlation by the developed models in stage 3. Supplementary Table S4 shows the completed details about the baseline (real labels) accuracy and average accuracy on randomized labels obtained during Y-randomizations. Upon comparing all the models built in stage 3, we could see that baseline (real labels) accuracy was higher (in moderate to higher ranges) in all cases than average accuracy on randomized labels. Specifically, baseline (real labels) accuracy and average accuracy on randomized labels for QDA + ROS; QDA + Undersampling; XGBClassifier + ROS; and XGBClassifier + Undersampling models were 0.9120 and 0.8272; 0.8611 and 0.7354; 0.8611 and 0.7331; and 0.9074 and 0.8233, respectively. The moderate to higher difference between the real accuracy and average accuracy on randomized labels suggested that the developed model did not have chance correlation and can be further used for classification.

**FIGURE 3:**
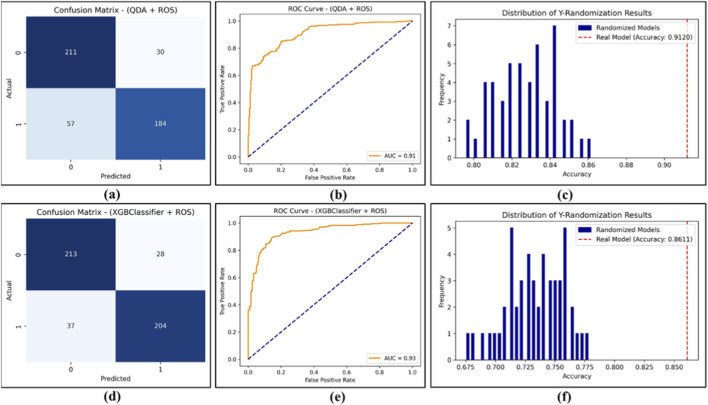
**(a–d)** Confusion matrix, **(b–e)** ROC-AUC curve, and **(c–f)** Y-randomization results for the top 2 models.

Finally, the top 2 robust models at stage 3 ML modelling were evaluated for the confusion matrix. Figure 3 shows the confusion matrix, ROC-AUC curve, and distribution of Y-randomization representations for QDA + ROS and XGBClassifier + ROS. It can be seen that both selected models had a higher number of true positives and true negatives, and fewer false positives and false negatives in the confusion matrix. This determined the robustness of the developed ML models. Similarly, the ROC values of 0.91 and 0.93 signified the robustness of the selected models. The developed models were saved using the Pickle module. These analyses made QDA + ROS and XGBClassifier + ROS suitable for further evaluation using recently used Explainable-Artificial Intelligence (XAI).

### 3.4 XAI using the SHAP framework

**FIGURE 4:**
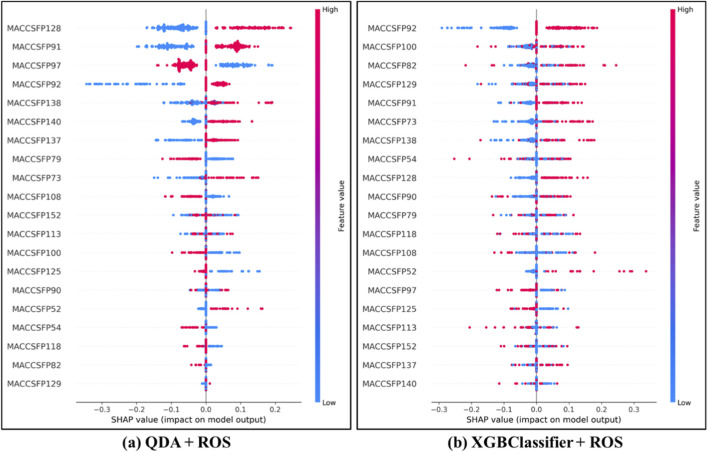
Explanation by XAI for **(a)** QDA + ROS and **(b)** XGBClassifier + ROS models.

The developed SIRT1-based ML models in stage 3 of ML modeling were further subjected to SHAP frameworks to address the black-box nature of QDA + ROS and XGBClassifier + ROS SIRT1-based ML models. The XGBoost classifier, a tree-based model, has been explained using TreeExplainer, while the non-tree-based QDA model has been explained using KernelExplainer according to guidelines in previous research studies (Lundberg et al., 2020). Figure 4 describes the XAI approach of QDA + ROS and XGBClassifier + ROS models. The Y-axis shows that the features are ranked by their average impact on the model, and the most important features for the model predictions were placed at the top. The X-axis represents the impact on the model’s output in terms of positive (right side) and negative (left side) values. The colour code red (high) showed the presence (1) of a MACCS feature (bit), whereas the blue colour showed the absence (0) of a MACCS feature (bit). Each dot in the plot, red or blue, represented a single molecule of the test dataset. In general, the features at the top of the beeswarm plot impact most, whereas the features at the bottom impact the least on the model’s decision-making capability. The positive values favoured the active prediction of the compound, whereas the negative values favoured the inactive prediction of the compound in the test data set. To identify the feature that favours active prediction of a compound, look for most of the red dots on the right-hand side, and the presence of such a feature in an unknown compound would increase its chances of being an active molecule. Similarly, the feature that has more red dots on the left-hand side of the plot would favour an inactive prediction of the compound. Blue and red dots mixed on both the left and right-hand side of the plot will reflect the compound context-dependent feature of the model, which sometimes favours and sometimes does not the activity of the compound. Lastly, the features that have all the dots near zero (bottom features) are the non-informative features for the model’s decision-making capabilities.

#### 3.4.1 SHAP framework analysis for QDA + ROS model

The QDA + ROS model covered the linear complexity of the dataset. The QDA + ROS model could be explained based on the SHAP beeswarm plot (Figure 4a) obtained through the SHAP framework. The features such as MACCSFP128, MACCSFP91, MACCSFP92, MACCSFP138, MACCSFP140, MACCSFP137, and MACCSFP73 were active prediction-favoring, as per the model, whereas features such as MACCSFP97, MACCSFP79, and MACCSFP108 were inactive, as per the model.

#### 3.4.2 SHAP framework analysis for XGBClassifier + ROS model

The XGBClassifier + ROS model, being a tree-based model, covered the non-linear complexity of the dataset. Based on the beeswarm plot of the XGBClassifier + ROS model (Figure 4b), MACCSFP92, MACCSFP100, MACCSFP82, MACCSFP129, MACCSFP91, MACCSFP73, MACCSFP138, MACCSFP128, and MACCSFP52 were the active prediction-favoring feature, whereas MACCSFP54, MACCSFP108, MACCSFP97, and MACCSFP113 the major inactive prediction-favoring feature as per the model. Some features were context-dependent on the same model.

The XAI analyses using the SHAP framework for QDA + ROS and XGBClassifier + ROS models reflected a common set of MACCS fingerprint features predictive of SIRT1 activity. For instance, MACCSFP92 (N surrounded by 2-heteroatoms), MACCSFP91 (Heteroatom sequence), MACCSFP128 (achiral or chiral center), MACCSFP138 [Double bond between C and N (C=N)], and MACCSFP73 (Sulfur environment) were all identified as robust key structural determinants predictive of active versus inactive SIRT1 activity in both models, indicating a strong correlation with the SIRT1 activity prediction output of both models. Compounds such as Sirtinol and Ex-527, known inhibitors of SIRT protein, contain “N” in their structure. This suggested the importance of the N-atom in SIRT1 active compounds. This N-atom might be contributing by acting as a hydrogen bond acceptor for key residues Asp348, etc., in the binding pocket of the SIRT1 protein. Similarly, a sulphur environment and chiral scaffolds may enable the attainment of a specific 3D orientation of the inhibitor molecule in the binding pocket of the SIRT1 protein.

**FIGURE 5:**
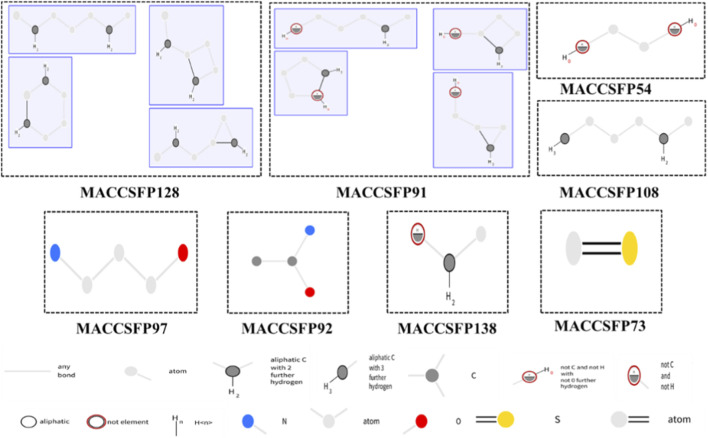
Structural arrangements of the common features obtained after XAI analysis of both models.

Conversely, MACCSFP54 (A longer heteroatom-hydrogen sequence), MACCSFP97 (N≡N or specific N patterns), and MACCSFP108 (aliphatic chain) were consistently associated with inactive SIRT1 activity predictions. A long aliphatic chain may suggest the inactive nature of the compound due to steric clashes or unfavorable binding energy in the binding pocket of the SIRT1 protein. While the QDA model exhibited directionally stable SAR across several compounds tested, the XGBClassifier also represented the ability to capture higher-order chemical complexity (interaction) that occurs due to the combination of these perceived stable SARs. Overall, these results suggest that SIRT1 activity prediction could be made based on a cooperative chemical fingerprint signature rather than with respect to local structural alerts alone, hence providing additional value in SAR-based screening and design to obtain novel SIRT1 modulators. The most important features in the XAI analyses were identified with RDKit as SMARTS annotations and visualized through SMARTSview in the SMARTSPLUS tool (Ehrt et al., 2020). The detailed representations of the most important features have been shown in Figure 5.

### 3.5 Applicability domain assessment

**FIGURE 6:**
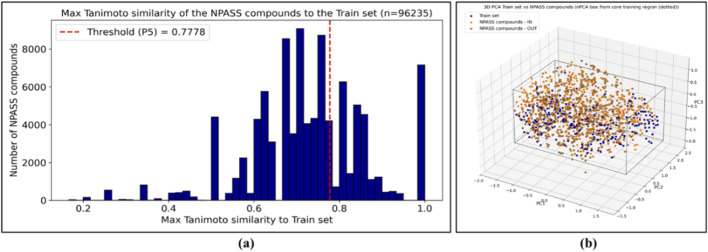
AD analysis **(a)** maximum neighbor Tanimoto similarity of the NPASS compound to the train set, **(b)** 3D PCA of the train set and the NPASS compounds.

The unknown natural compounds (96,235) were retrieved from the NPASS database, followed by the generation of MACCSFP and the retention of the same top 20 features as those used in model building. Before using SIRT1-based ML models (QDA + ROS and XGBClassifier + ROS), the applicability domain (AD) of the unknown database was employed to assess the reliability in an external chemical space using the nearest neighbor Tanimoto similarity and 3D PCA methods. The step also helped to ensure the model’s generalizability. Figure 6a shows the distribution of maximum Tanimoto similarity values for the closest neighbours of each NPASS compound in the training set. A threshold was kept at the fifth percentile of the training-set similarity distribution, which corresponds to a Tanimoto similarity value of 0.7778. Consequently, NPASS compounds that had similarities above 0.7778 were within the AD and labelled as (IN), and NPASS Compounds with similarities less than this threshold were labelled as outside the AD (OUT). This quantitative overlap of the train set, and the unknown compounds signified the structurally robust and sufficiently generalized predictions to the unknown chemical space. There was a total of 30,846 compounds (NPASS compound–IN), and the remaining one was NPASS compound–OUT. The resulting NPASS compound–IN exhibited high chemical similarity to the train set and good prediction reliability as a result of the AD barrier executed in this stage. A 3D PCA visualization and AD verification of the entire NPASS data, which included the train set (blue), NPASS compound–IN (orange without black boundary), and NPASS compound–OUT (orange with black boundary), was made by utilizing the specified MACCS fingerprints. The resulting plot is shown in Figure 6b. The 3D PCA plot indicated that the majority of NPASS compound -IN clustered within the same chemical region as the training set and most frequently appeared in the center of the training set’s region. In contrast, NPASS-OUT compounds displayed a large difference to the training set because most NPASS-OUT compounds were located outside the Convex hull formed around the training PCA space.

### 3.6 Prediction of the NPASS compounds

Around 30,846 compounds were obtained as NAPSS compounds - IN after AD analysis. These compounds were further considered for the classification as active (1) or inactive using previously validated QDA + ROS and XGBClassifier + ROS, SIRT1-based ML models. From the QDA + ROS model total of 16,166 compounds were predicted to be active, remaining inactive, whereas XGBClassifier + ROS SIRT1-based ML models provided 2,360 compounds as active. Ultimately, a total of 943 active compounds were found from QDA + ROS and XGBClassifier + ROS models. These commonly active predicted compounds were considered for the next stages.

### 3.7 Primary level virtual screening using UniDock

For the initial screening, the 943 compounds were subjected to ADMET-AI with relaxed constraints as specified earlier. A total of 480 natural compounds crossed this stage. All 480 compounds were further carried out for primary level virtual screening using UniDock. Top 30% of the compounds, i.e., 144 compounds, were considered for the next stage. This threshold was picked to avoid exclusion of the potentially active compound in the early drug screening.

### 3.8 Secondary level virtual screening using AutoDock-GPU

A total of 144 compounds were subjected to secondary-level virtual screening using AutoDock-GPU. Unfortunately, few compounds exhibited non-standard elements that were not recognized by the AutoDock-GPU tool. We excluded such compounds to yield 120 compounds for secondary-level virtual screening. This stage was facilitated by 100 poses of each natural compound in the binding pocket of the SIRT1 protein. The top 30% of the input compounds were shortlisted for further rigorous ADMET analysis.

### 3.9 ADMET using SWISSADME and the Protox tool

A total of 36 compounds crossed the secondary level of virtual screening. These compounds were further evaluated with the SwissADME tool. The filters used were GI absorption, Lipinski, and PAINS. Out of 36 compounds, 11 compounds crossed the ADME barrier. These 11 compounds were further considered for toxicity (hepatotoxicity, carcinogenicity, immunotoxicity, mutagenicity, and cytotoxicity) analysis. We found four compounds that were completely non-toxic with all the above-mentioned filters. The detailed information of the 11 compounds is mentioned in Supplementary Table S5. The selected four compounds were NPC216682 (PubChem ID: 163189091), NPC480509 (PubChem ID: 4891), NPC210910 (PubChem ID: 163093125), and NPC247082 (PubChem ID: 10641320). These four top hits bound with the SIRT1 protein were thoroughly investigated in post-docking interaction analysis in DSV software.

### 3.10 Post-docking interactions analysis

The top four hits, as mentioned in the previous step, were deeply explored using DSV. Table 1 describes the completed information about all four hits and the reference compound found in the study, their docking scores, key interactions forming residues, binding pockets, and 2D and 3D interactions images.

**TABLE 1:** AutoDock-GPU-based docking score (kcal/mol) and interactions profile of the docked complexes.

NPASS ID	Name	Autodock-GPU scores	Conventional H-bonds and C-H bond	Binding pocket
NPC216682	1-[5-[(1S,2R,8S,9R,10S)-7,15-diazatetracyclo[7.7.1.02,7.010,15] heptadecan-8-yl]-3,4-dihydro-2H-pyridin-1-yl]ethanone	−11.09	ALA262, VAL412	GLY261, ALA262, ASP272, PHE273, ARG274, PHE297, GLN345, ASN346, ILE347, HIS363, PHE413, PHE414, GLY440, SER441, VAL445
NPC480509	Praziquantel	−10.22	ARG274, PHE297	GLY261, ALA262, PHE273, PHE297, ILE316, GLN345, ASN346, ILE347, HIS363, VAL411, VAL412, PHE413, PHE414, SER441, VAL445
NPC210910	2-[2-[(2S)-5-amino-3,4-dihydro-2H-pyrrol-2-yl]-4-oxoquinazolin-3-yl]benzoic acid	−9.81	ARG274, VAL412 (3)	PHE273, PHE297, GLN345, HIS363, VAL445
NPC247082	2,2-Bis(3,3′-indolyl)-isocaproic acid	−9.71	PHE297, GLN345, HIS363	PHE273, ARG274, PHE297, ASP298, ILE316, ASN346, ILE347, HIS363, ILE411, VAL412, PHE413, PHE414, VAL445
Reference	(6S)-2-Chloro-5,6,7,8,9,10-hexahydro-cyclohept[b]indole-6-carboxamide	−8.54	GLN345, VAL412	ALA262, ILE270, PHE273, PHE297, ILE316, ASN346, ILE347, HIS363, ILE411, PHE413

The reference compound, i.e., the co-crystallized ligand ((6S)-2-Chloro-5,6,7,8,9,10-hexahydro-cyclohept[b]indole-6-carboxamide) in the binding pocket of the SIRT1 protein attained a docking score of −8.54 kcal/mol. The conventional H-bonds and C-H bonds were GLN345 and VAL412. The other residues that were involved in the binding pocket were ALA262, ILE270, PHE273, PHE297, ILE316, ASN346, ILE347, HIS363, ILE411, and PHE413. The position of the reference ligand in the binding pocket of the SIRT1 protein was similar to that previously reported in the literature (Sinha et al., 2019; Ullah et al., 2023). This observation marked the significance of our docking protocol and also provided a strong reason to compare with other hit compounds.

**FIGURE 7:**
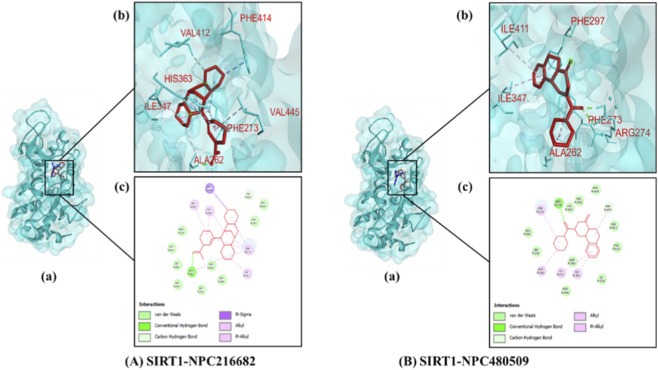
Representation of (a) superimposition of hit (red) with co-crystallized ligand (reference) (blue), (b) 3D interactions, and (c) 2D interactions of **(A)** SIRT1-NPC216682 and **(B)** SIRT1-NPC480509 complexes.

The first hit compound was NPC216682, which has the IPAC name as 1-[5-[(1S,2R,8S,9R,10S)-7,15-diazatetracyclo [7.7.1.02,7.010,15] heptadecan-8-yl]-3,4-dihydro-2H-pyridin-1-yl] ethenone. NPC216682 exhibited a docking score of −11.09 kcal/mol. The conventional H-bonds and C-H bonds were formed with ALA262 and VAL412. The other residues in the binding of the SIRT1-NPC216682 complex were GLY261, ALA262, ASP272, PHE273, ARG274, PHE297, GLN345, ASN346, ILE347, HIS363, PHE413, PHE414, GLY440, SER441, and VAL445 (Figure 7A). The binding of NPC216682 resembled that of the reference compound, and the lower docking scores marked the significance of this hit compound. This hit followed all the ADMET profiles considered in this study. The probability of being an active molecule by QDA + ROS and XGBClassifier + ROS was found to be 0.99 and 0.50, respectively. NPC216682 can be found in plant sources such as *Tephrosia tinctoria*, *Allanblackia stuhlmannii*, *Boehmeria caudata*, *Chaenomeles japonica*, etc. NPC216682 belongs to the class of organic compounds, as Quinolizidine alkaloids. Specifically, this hit contains two fused piperidine rings and one acetylated piperidine ring. The overall structure of this hit is that of a dense, non-aromatic, nitrogen-containing heterocyclic compound (Figure 9). This Hit’s structural composition is more favored as per the QDA + ROS model preferences. Therefore, it was predicted to be active with greater active prediction confidence. Unfortunately, no specific activity of the compound has been reported so far. This marked the novelty and scope of this study.

Another hit of this study was NPC480509, which has a common Praziquantel. This Hit attained a docking score of −10.22 kcal/mol upon forming conventional H-bonds or C-H bonds with residues ARG274 and PHE297. Though the key residues involved in the formation of the H-bond were not similar to the reference compound, the docking score difference to that of the reference compound was significant. This might be achieved due to other residues present in the binding pocket, such as GLY261, ALA262, PHE273, PHE297, ILE316, GLN345, ASN346, ILE347, HIS363, VAL411, VAL412, PHE413, PHE414, SER441, and VAL445 (Figure 7B). NPC480509 followed the selected ADMET parameter to a good extent. The active prediction confidence for this hit was estimated as 0.99 and 0.82. Interestingly, this hit was predicted as active with high probabilities by both models. The source of praziquantel is mainly the *Baccharis lateralis* plant, and it has been widely used for its medicinal uses, such as anthelmintics (anti-parasitic) and cytotoxic agents against cancer cells (Budel et al., 2018; Sessa et al., 2020). Praziquantel comes in the class of tetrahydroisoquinolines. The hit compound praziquantel consists of a six-membered ring containing two nitrogen atoms and a carbonyl group (piperazinone ring) in the centre. This central ring is attached to tetrahydroisoquinoline (a benzene ring fused to a saturated six-membered nitrogen-containing ring). On the other side of the piperazinone ring, an acetyl benzene group is also attached to form the complete structure of praziquantel (Figure 9). Praziquantel exhibits a synergistic property that enhances the anti-tumor effect of the cytotoxic drug paclitaxel on the proliferation of various malignant tumors, affecting cancer cell lines by inducing both mitotic arrest and apoptosis (Wu et al., 2012).

**FIGURE 8:**
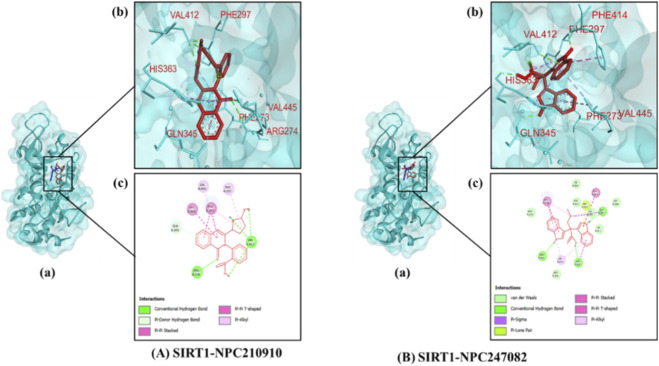
Representation of (a) superimposition of hit (red) with co-crystallized ligand (reference) (blue), (b) 3D interactions, and (c) 2D interactions of **(A)** SIRT1-NPC210910 and **(B)** SIRT1-NPC247082 complexes.

**FIGURE 9:**
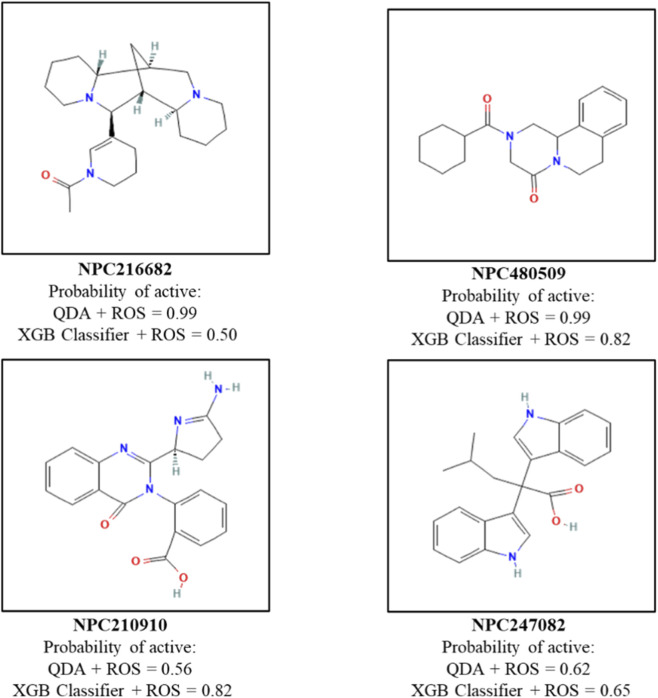
Structural representation of the top four identified compounds for SIRT1 predicted inhibition.

NPC210910 was the next hit obtained, which has the IUPAC name as 2-[2-[(2S)-5-amino-3,4-dihydro-2H-pyrrol-2-yl]-4-oxoquinazolin-3-yl] benzoic acid, and a docking score of −9.81 kcal/mol. There were four conventional H-bonds and C-H bonds with residues ARG274 and VAL412. Other residues involved in the binding pocket were PHE273, PHE297, GLN345, HIS363, and VAL445 (Figure 8A). NPC210910 followed all ADMET barriers used in the study. This hit was predicted to be completely non-toxic. The probability of active was identified as 0.56 and 0.82 by QDA + ROS and XGBClassifier + ROS models. The main plant and non-plant sources of this hit are *Necturus maculosus*, *Melolontha vulgaris*, *Licaria rigida*, *Haplophyton cimicidum*, etc. It comes in the class of quinazolines. The hit compound is composed of oxoquinozolin (Quinazolinone) as one moiety. An amino-substituted dihydropyrrole ring at the top right of the oxoquinozolin ring. Benzoic acid as another substituent is also present to nitrogen atom of the oxoquinozolin ring to form the complete structure. Overall, this hit is aromatic due to its greater richness in heterocycles (Figure 9). This explained the high active prediction confidence of XGBClassifier + ROS model to the NPC210910. Unfortunately, no activity of this compound has been reported in the literature so far.

Lastly, compound NPC247082 was another hit identified in this study which has the IUPAC name of 2,2-Bis(3,3′-indolyl)-isocaproic acid. NPC247082 exhibited a docking score of −9.71 kcal/mol upon forming conventional H-bonds and C-H bonds with residues PHE297, GLN345, and HIS363, along with other residues, such as PHE273, ARG274, PHE297, ASP298, ILE316, ASN346, ILE347, HIS363, ILE411, VAL412, PHE413, PHE414, and VAL445 involved in the binding pocket of the SIRT1 protein (Figure 8B). This hit compound also followed all the ADMET parameters, which predicted the clinical significance of the predicted hit compound. The active prediction confidence was noted as 0.62 and 0.65 by QDA + ROS and XGBClassifier + ROS models. The main source of this hit is the *Beilschmiedia zenkeri* plant. It comes in the class of indoles and derivatives under tryptophan alkaloids. The hit compound belongs to the bis-indole family. In the second position, isocaproic acid is substituted by two indole groups to form the complete structure of this Hit compound (Figure 9). Here, no specific activity has been proven so far. This marked the scope of the study.

Upon comparing the molecular docking outcomes of the top four hits, we could say that all four hits attained lower docking scores than the reference compound, along with the formation of similar interactions and larger binding pockets. All these four hits were further evaluated in dynamic environments and compared with the reference compound and the SIRT1 apoprotein.

### 3.11 Molecular dynamics simulation studies

The molecular docking allowed only the ligand to be in a dynamic state, whereas the SIRT1 protein was rigid. The complete understanding of the docked complex was done using molecular dynamics simulations (MDS), where both ligand and proteins were allowed to move in a setting of periodic boundary conditions. SIRT1 protein in the apo state, and SIRT1 protein bound with reference, NPC216682, NPC480509, NPC210910, and NPC247082 compounds were considered for a 200 ns MDS study in the GROMACS 24.3 software. The completion of each trajectory was followed by rigorous evaluations such as RMSD, RMSF, RG, SASA, H-bonds analysis, H-bond % occupancy analysis, PCA, and FEL plots.

**FIGURE 10:**
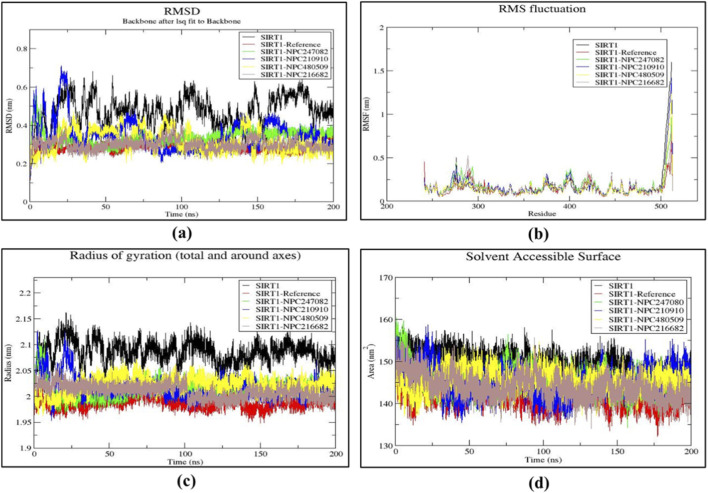
Representation of **(a)** RMSD, **(b)** RMSF, **(c)** RG, and **(d)** SASA for SIRT1 (black), SIRT1-Reference (red), SIRT1-NPC247082 (green), SIRT1-NPC210910 (blue), SIRT1-NPC480509 (yellow), and SIRT1-NPC216682 (brown).

The RMSD values signify the change in the position of protein atoms from the initial state. In general, lower RMSD values refer to a stable system. In our study, the SIRT1 protein in the apo state was found to be highly unstable. Many major and minor fluctuations were observed within this system. In contrast, the SIRT1 protein bound with the reference and other hit compounds attained a very stable state throughout the simulation period. The overall average RMSD values for the selected six systems were found as 0.4715 ± 0.0793 nm, 0.2873 ± 0.0232 nm, 0.3372 ± 0.0370 nm, 0.3486 ± 0.0807 nm, 0.3157 ± 0.0596 nm, and 0.2985 ± 0.0287 nm for SIRT1, SIRT1-Reference, SIRT1-NPC247082, SIRT1-NPC210910, SIRT1-NPC480509, and SIRT1-NPC216682, respectively (Table 2). In Figure 10a, SIRT1 bound to the reference compound, NPC216682, and NPC480509 were highly stable. However, some minor fluctuations were present in the SIRT1-NCP480509 complex. The trajectories for the complexes like SIRT1-NPC247082 and SIRT1-NPC210910 fluctuated at the beginning (20–25 ns) of MDS, but they stabilized further. Based on RSMD analysis, the order of stability could be depicted as SIRT1-NPC216682, SIRT1-NPC480509, and the SIRT1-reference compound, which were highly stable with close average RMSD values; the complexes SIRT1-NPC210910 and SIRT1-NPC247082 were moderately stable, and SIRT1 alone was highly unstable.

**TABLE 2:** Average RMSD, RMSF, Rg, SASA, H-bond for SIRT1, SIRT1-Reference, SIRT1-NPC247082, SIRT1-NPC210910, SIRT1-NPC480509, and SIRT1-NPC216682.

Complex	RMSD (nm)	RMSF (nm)	Rg (nm)	SASA (nm^2^)	H-bonds
SIRT1	0.4715 ± 0.0793	0.1709 ± 0.1859	2.08 ± 0.0205	149.32 ± 2.8109	-
SIRT1-Reference	0.2873 ± 0.0232	0.1442 ± 0.0794	1.9887 ± 0.0109	142.02 ± 2.5131	0–8
SIRT1-NPC247082	0.3372 ± 0.0370	0.1793 ± 0.1094	2.01 ± 0.0166	145.31 ± 2.9542	0–5
SIRT1-NPC210910	0.3486 ± 0.0807	0.1764 ± 0.1581	2.01 ± 0.0205	145.17 ± 3.4354	0–6
SIRT1-NPC480509	0.3157 ± 0.0596	0.1537 ± 0.1300	2.02 ± 0.0158	145.43 ± 2.5165	0–4
SIRT1-NPC216682	0.2985 ± 0.0287	0.1630 ± 0.0948	2.00 ± 0.0135	143.93 ± 3.0396	0–4

The RMSF analysis defines the fluctuations of each residue throughout the simulation period. The lower RMSF values, along with the least fluctuations, resemble a stable system. All the system’s RMSF fluctuations were within 0.5 nm. The average RMSF values of SIRT1, SIRT1-Reference, SIRT1-NPC247082, SIRT1-NPC210910, SIRT1-NPC480509, and SIRT1-NPC216682 were calculated as 0.1709 ± 0.1859 nm, 0.1442 ± 0.0794 nm, 0.1793 ± 0.1094 nm, 0.1764 ± 0.1581 nm, 0.1537 ± 0.1300 nm, and 0.1630 ± 0.0948 nm, respectively (Table 2). Based on average RMSF values and the trend in the RMSF plots (Figure 10b), we could say that the systems in the order of stability, as SIRT1-reference, SIRT1-NPC480509, and SIRT1-NPC216682, were the most stable complexes, and SIRT1-NPC247082, SIRT1-NPC210910, and SIRT1 alone resembled each other’s RMSF plots.

The RG analysis describes the compactness of a system around its central axis. Generally, a lower RG value defines a compact and stable system. In Figure 10c, SIRT1 in apo state attained a highly fluctuating RG trajectory, whereas in the ligand-bound state these fluctuations were primarily reduced. However, the average RG values of were found to be close enough as 2.08 ± 0.0205 nm, 1.98 ± 0.0109 nm, 2.01 ± 0.0166 nm, 2.01 ± 0.0205 nm, 2.02 ± 0.0158 nm, and 2.00 ± 0.0135 nm for SIRT1, SIRT1-Reference, SIRT1-NPC247082, SIRT1-NPC210910, SIRT1-NPC480509, and SIRT1-NPC216682, respectively (Table 2). The order of stability based on RG analysis could be defined as SIRT1-Reference, SIRT1-NPC480509, and SIRT1-NPC216682 being equally stable complexes, and SIRT1-NPC247082, and SIRT1-NPC210910 being moderately stable complexes.

The SASA analysis measures the surface area of protein that is exposed to the solvent. Generally, a lower SASA value defines a stable system. In Figure 10d, most of the SASA trajectories are overlapping. The average SASA values were calculated as 149.32 ± 2.8109 nm^2^, 142.02 ± 2.5131 nm^2^, 145.31 ± 2.9542 nm^2^, 145.17 ± 3.4354 nm^2^, 145.43 ± 2.5165 nm^2^, and 143.93 ± 3.0396 nm^2^ for SIRT1, SIRT1-Reference, SIRT1-NPC247082, SIRT1-NPC210910, SIRT1-NPC480509, and SIRT1-NPC216682, respectively (Table 2). Based on the SASA analysis, the SIRT1 in the apo state was considered as a highly unstable system, whereas the order of the ligand-bound state was observed as SIRT1-Reference being most stable, followed by SIRT1-NPC216682, SIRT1-NPC480509, SIRT1-NPC210910, and SIRT1-NPC247082. However, the average SASA values of SIRT1-NPC480509, SIRT1-NPC210910, and SIRT1-NPC247082 were close enough.

H-bond analysis dominantly defines the stability of a protein-ligand system. A larger number of H-bonds indicates more stability. In this study, the number of H-bonds identified was 0–8, 0–5, 0–6, 0–4, and 0–4 for SIRT1-Reference, SIRT1-NPC247082, SIRT1-NPC210910, SIRT1-NPC480509, and SIRT1-NPC216682 complexes, respectively (Table 2). Here, a larger number of H-bonds and high stability of the reference compound in the binding pocket of the SIRT1 protein. The order of stability of the hit compounds based on H-bond analysis was SIRT1-NPC210910 being the most stable, followed by SIRT1-NPC247082, SIRT1-NPC480509, and SIRT1-NPC216682. Notably, the trend in the stability of the hit compound did not match that of other analyses. The possible reason could be the involvement of other interactions (hydrophobic) in the stability of the SIRT1-NPC480509 and SIRT1-NPC216682 complexes.

**FIGURE 11:**
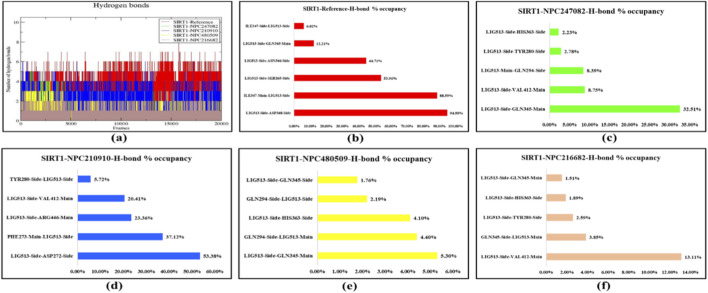
Representation of **(a)** formation of number H-bonds; donor-acceptor vs. H-bond % occupancy plot for **(b)** Reference, **(c)** NPC247082, **(d)** NPC210910, **(e)** NPC480509, and **(f)** NPC216682.

In Figures 11b–f, the plots of percentage (%) occupancy against donor-acceptor pairs at 3.5 Å distance and 30^◦^ angle, throughout the simulation period. In the SIRT1-Reference complex, formed H-bond with ASP348 (94.95%), ILE347 (88.59%), SER265 (53.96%), ASN365 (44.71%), GLN345 (12.21%), and ILE347 (6.02%). In the SIRT1-NPC247082 complex, the H-bond % occupancy was seen as GLN345 (32.51%), VAL412 (8.75%), GLN294 (8.39%), TYR280 (2.78%), and HIS363 (2.23%). In the SIRT1-NPC210910 complex, the H-bond % occupancy was seen with residues ASP272 (53.38%), PHE273 (37.12%), ARG446 (23.36%), VAL412 (20.41%), and TYR280 (5.72%). In the SIRT1-NPC480509 complex, the H-bond % occupancy occurred with respect to GLN345 (5.30%), GLN294 (4.40%), HIS363 (4.10%), GLN294 (2.19%), and GLN345 (1.76%). Lastly, in the SIRT1-NPC216682 complex, the H-bond % occupancy was seen with residues VAL412 (13.11%), GLN345 (3.85%), TYR280 (2.59%), HIS363 (1.89%), and GL345 (1.51%).

Overall, the trends in the formation of H-bonds or the % occupancy of H-bond is more favorable for NPC247082 and NPC210910 compounds. Specifically, a similar residue (GLN345) as that of the reference compound, along with other key residues, was involved in the stabilization of the SIRT1-NPC247082 complex. This marked the significance of the NPC247082 hit compound. In the SIRT1-NPC210910 complex, a high % of H-bond occupancy was also achieved, but with different residues than those of the reference compound. We could say the stabilization of both NPC247082 and NPC210910 compounds in the binding pocket of the SIRT1 protein was due to the H-bond formation. In contrast, SIRT1-NPC480509 and SIRT1-NPC216682 showed low % H-bond occupancy values. And that also included some slightly similar residues to those of the reference compound. Based on this observation, we could conclude that the SIRT1-NPC480509 and SIRT1-NPC216682 complexes were stabilized more by other hydrophobic interactions, driven by key residues in the binding pocket, than by H-bonds. This justified the previous observation trends as well as the selectivity of the identified compound in the binding pocket of the SIRT1 protein.

**FIGURE 12:**
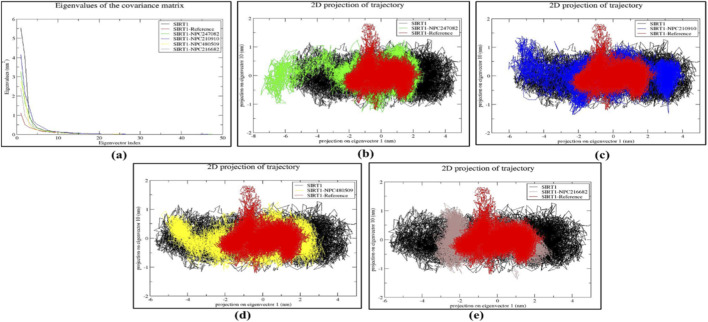
Representation of **(a)** covariance matrix, **(b–e)** PCA for SIRT1 (black), SIRT1-Reference (red), SIRT1-NPC247082 (green), SIRT1-NPC210910 (blue), SIRT1-NPC480509 (yellow), and SIRT1-NPC216682 (brown).

The linear combination of atomic motions, known as an eigenvector, describes the overall movement of the protein. These atomic motions represent biological activity and deformation patterns (Jena et al., 2025). Therefore, PCA studies were performed to identify the eigenvectors involved in the global motion of the C-alpha atoms of the protein in apo form and holo form. The global motion of the protein is mainly because of the first few eigenvectors of the protein. The digitalization of the matrix was performed to identify such eigenvectors. Importantly, a low eigenvalue with a high eigenvector shows a less dynamic and more stable system. In Figure 12a, it can be seen that the reference compound, followed by NPC216682 and NPC480509, bound with the SIRT1 protein had the lowest eigenvalues with higher eigenvectors than other systems. This determined the less dynamic motion in the aforementioned systems. The other hit compounds, NPC247082 and NPC210910, bound with the SIRT1 protein attained moderate but better than the SIRT1 apo form dynamic nature throughout the simulation period.

**FIGURE 13:**
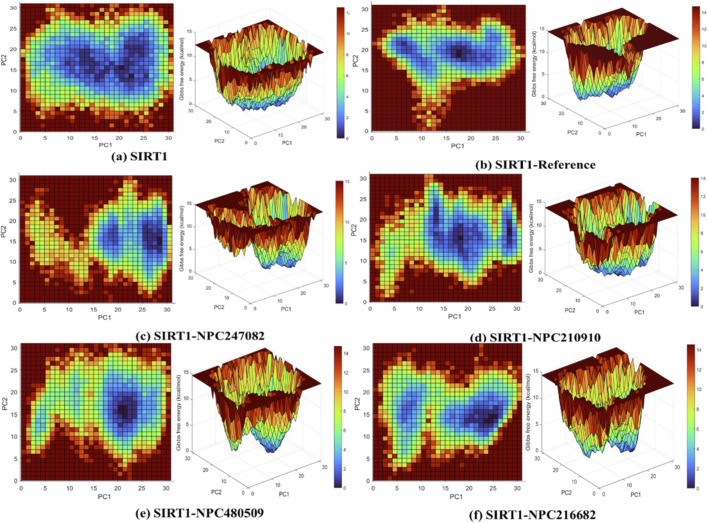
2D and 3D FEL plots of **(a)** SIRT1, **(b)** SIRT1-Reference, **(c)** SIRT1-NPC247082, **(d)** SIRT1-NPC210910, **(e)** SIRT1-NPC480509, and **(f)** SIRT1-NPC216682.

The 2D PCA plots were aligned with the observation of the covariance matrix. SIRT1 in apo form attained a large conformational space, which stated its most dynamic nature among other systems. In contrast, the SIRT1-Reference complex attained the least conformational space compared to all the systems, which marked the least dynamic nature of the reference compound in the binding pocket of the SIRT1 protein. Upon comparing the 2D PCA plots of the hits compound, it could be found that NPC216682 acquired the least conformational space and maximum stability, followed by NPC480509, NPC247082, and NPC210910 in the binding pocket of the SIRT1 protein.

The FEL plots were also made to understand the stability of each system with respect to energy content. An FEL plot provides a graphical depiction of the minimum energy conformation found within a protein-ligand complex. A deep blue region corresponds to a stable region, whereas a red region corresponds to an unstable region. An unstable system interaction formed between a weakly interacting compound produces several clusters of minimum energy conformations (deep blue regions); conversely, a strongly interacting protein-ligand system will generate fewer than three clusters representing the single most stable conformation of the system. In the present study, the Gibbs free energy for SIRT1, SIRT1-Reference, SIRT1-NPC247082, SIRT1-NPC210910, SIRT1-NPC480509, and SIRT1-NPC216682 were calculated to be 12.5 kcal/mol, 14.7 kcal/mol, 15.0 kcal/mol, 14.0 kcal/mol, 14.7 kcal/mol, and 14.5 kcal/mol, respectively. SIRT1 in apo form exhibited a highly scattered deep blue region. This indicates that no specific stable conformation was achieved in the SIRT1 apo form. The reference compound, upon binding to the SIRT1 protein, stabilized the protein to some extent. SIRT1-Reference complex attained two minimum energy blue regions. Upon comparing to the hit compounds, NPC247082 and NPC480509 stabilized the SIRT1 protein maximum, followed by NPC216682 and NPC210910 according to FEL analysis (Figure 13).

### 3.12 MMPBSA calculation

The selected protein ligand system was analyzed for MMPBSA calculation. The total binding free energy was calculated for SIRT1-Reference, SIRT1-NPC247082, SIRT1-NPC210910, SIRT1-NPC480509, and SIRT1-NPC216682 to be (−31.77 ± 2.92 kcal/mol), (−14.56 ± 1.82 kcal/mol), (−23.74 ± 2.47 kcal/mol), (−21.39 ± 2.23 kcal/mol), and (−32.52 ± 2.60 kcal/mol), respectively (Table 3). Based on MMPBBSA calculations, we could predict the greater stability of the SIRT1-NPC216682 complex than the SIRT1-Reference complex. The other complexes’ stability could be ordered as SIRT1-NPC210910 and SIRT1-NPC480509, moderately stable, and SIRT1-NPC247082 being the least stable complex.

**TABLE 3:** MMPBSA calculations, where: ΔVDWAALS: Van der Waals contribution, ΔEEL: electrostatic energy, ΔEGB: polar solvation free energy, ΔESURF: nonpolar solvation free energy, ΔGGAS: gas face free energy, ΔGSOLV: solvation energy, ΔTOTAL: total calculated free binding energy, i.e., ΔGBinding (all units are reported in kcal/mol).

Energy component (kcal/mol)	ΔVDWAALS	ΔEEL	ΔEGB	ΔESURF	ΔGGAS	ΔGSOLV	ΔTOTAL
SIRT1-Reference	−26.58 ± 2.86	−35.68 ± 3.98	34.50 ± 2.03	−4.01 ± 0.19	−62.27 ± 3.51	30.49 ± 2.01	−31.77 ± 2.92
SIRT1-NPC247082	−31.61 ± 1.71	−6.89 ± 2.01	28.55 ± 2.06	−4.61 ± 0.21	−38.50 ± 2.91	23.94 ± 1.95	−14.56 ± 1.82
SIRT1-NPC210910	−29.63 ± 2.37	−20.64 ± 4.83	30.49 ± 3.95	−3.96 ± 0.22	−50.27 ± 5.07	26.53 ± 3.86	−23.74 ± 2.47
SIRT1-NPC480509	−33.98 ± 2.23	−12.08 ± 3.28	29.11 ± 3.32	−4.44 ± 0.27	−46.06 ± 4.39	24.67 ± 3.16	−21.39 ± 2.23
SIRT1-NPC216682	−46.95 ± 2.22	−6.76 ± 2.35	26.89 ± 2.44	−5.69 ± 0.25	−53.72 ± 3.39	21.20 ± 2.35	−32.52 ± 2.60

### 3.13 MTT assay

Among the identified compounds, hit compounds NPC216682 and NPC480509 exhibited higher stabilization, whereas NPC210910 and NPC247082 showed moderate stabilization. As described previously, among these compounds, NPC480509 (Praziquantel) has been reported for its anticancer properties, along with major anti-helminthic applications (Budel et al., 2018; Sessa et al., 2020). Also, the Praziquantel compound was the only commercially available compound that exhibited a strong active predicted probability by both SIRT1-based ML models. Therefore, we have procured the Praziquantel compound for the preliminary experimental validation via cell viability assay (MTT assay) on different human breast cancer cell lines (MDA-MB-231 and MCF7) and human normal breast cell line (MCF10A). The treatment of the test compound on breast cancer cell lines will allow us to comment on the anticancer properties of the identified compound, whereas normal breast cells will help us to know its non-toxic nature. The MTT assay was performed as described in methodology with five different concentrations of the test compound (12.5 μg/mL, 25 μg/mL, 50 μg/mL, 100 μg/mL, and 200 μg/mL) and one group as a negative control, which contained only media and the cell lines. The test compound exhibited a dose-dependent % cell inhibition on breast cancer cell lines. In MDA-MB-231 TNBC cells, the average absorbance values reduced from 0.474 at 12.5 μg/mL to 0.132 at 200 μg/mL, compared to the control value of 0.566. This reduction in average absorbance values depicted the increase in the cell inhibition from 16.1% at 12.5 μg/mL to 76.63% at 200 μg/mL. The calculated IC_50_ value was 59.66 μg/mL with a strong correlation between concentration and inhibition (*R* ^2^ = 0.999).

Similarly, in non-TNBC cell lines, i.e., MCF7 cell line, the average absorbance values reduced from 0.434 at 12.5 μg/mL to 0.057 at 200 μg/mL compared with the control having the absorbance value of 0.524. The percentage of cell inhibition increased from 17.01% at 12.5 μg/mL to 89.29% at 200 μg/mL, which indicated a strong cytotoxic effect in this breast cancer cell line. The calculated IC_50_ value was found to be 38.45 μg/mL, with a high correlation coefficient (*R* ^2^ = 0.992).

In contrast, the test compound exhibited minimal cytotoxicity towards the normal breast cell line (MCF10A). The average absorbance values remained relatively constant (0.416–0.392), along with cell viability attaining above 92% even at the highest concentration of 200 μg/mL. These findings suggest that praziquantel exhibits minimal harm to normal cells while specifically inhibiting the growth of breast cancer cells.

**FIGURE 14:**
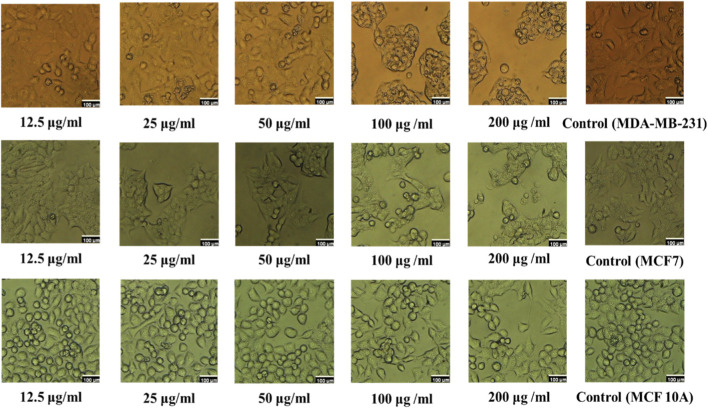
Microscopic images of MDA-MB-231, MCF7, and MCF10A cell lines treated with Praziquantel (NPC480509) in different concentrations, along with a negative control, and percentage growth inhibition from MTT assay.

**FIGURE 15:**
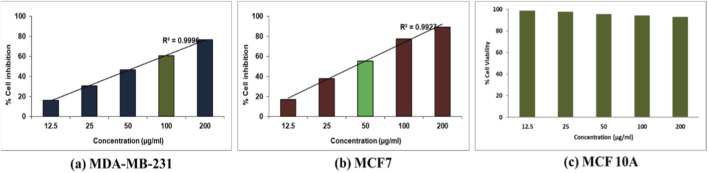
Results of MTT assay and IC_50_ value estimation by screening the percentage of cell inhibition in **(a)** MDA-MB-231, **(b)** MCF7, and **(c)** MCF10A cells upon treatment with Praziquantel (NPC480509) in different concentrations.

Overall, the MTT assay shows that praziquantel has moderate but specific cytotoxic activity against breast cancer cells, with MCF-7 cells showing a greater inhibitory effect than MDA-MB-231 cells (Figures 14, 15; Supplementary Tables S6, S7). However, gene expression studies using qRT-PCR and Western blotting to address the expression of the SIRT1 gene or protein are further suggested. Furthermore, advances in *in-vitro* and *in-vivo* can reveal the exact mechanism of action of Praziquantel on these breast cancer cell lines.

## 4 Discussion

Triple Negative Breast cancer (TNBC) exhibits the absence of hormone receptors, such as Estrogen Receptor (ER), Progesterone Receptor (PR), and Human Epidermal Growth Factor Receptor 2 (HER2), and accounts for the highest proportion, ranging from 6.7% to 27.9% worldwide (Sandhu et al., 2016; Kulkarni et al., 2020). The worrying nature of TNBC is further highlighted by reports of the highest proportions in India (Thakur et al., 2018). The absence of hormone receptors causes ineffective implications of conventional therapeutic approaches, like cytotoxic chemotherapy. Other constraints, such as high recurrence rates, off-target effects, and therapeutic resistance, demand new alternatives to tackle TNBC (Yin et al., 2020). Sirtuin1 (SIRT1) is a pronounced epigenetic modifier that deacetylates many oncogenes and tumor suppressors, making it a suitable target to cooperate with TNBC management (Rahman and Islam, 2011).

In a nutshell, this study aims to identify a natural compound inhibitor of the SIRT1 protein that can regulate the inactivation of tumor suppressors through the deacetylation process. To identify such a compound, a deep exploration of computational techniques has been utilized in this study. The collection of SIRT1-based compounds, input data preparation for ML modelling, three stages of ML modelling, explanation of the developed ML model (QDA + ROS and XGBClassifier + ROS) using SHapley Additive exPlanations (SHAP) framework [explainable AI (XAI)], applicability domain implication to NPASS library of natural compounds, and finally classification of an unknown dataset as active or inactive. We obtained a total of 943 compounds that were predicted to be active by both models, from moderate to high levels of confidence. These predicted active compounds were further taken for primary level virtual screening, secondary level virtual screening, and ADMET predictions. This part of the study was initially designed to identify a potential hit compound in the binding pocket of the SIRT1 protein through molecular docking. Four compounds (NPC216682, NPC480509, NPC210910, and NPC247082) were considered for molecular dynamics simulation (MDS), and the dynamic nature of the selected hits in the SIRT1 bound state was analysed with numerous trajectory analyses. After analysing Root mean square deviation (RMSD) of backbone residues, Root mean square fluctuations (RMSF), Radius of gyration (Rg), Solvent accessible surface area (SASA), and the number of Hydrogen bonds, Principal Component Analysis (PCA), and Free Energy Landscape (FEL), we estimated the stability, flexibility, selectivity, and conformational behaviour of the selected protein-ligand complexes. Finally, the total binding free energy calculation (MMPBSA) provided the quantitative estimation of binding affinity. We identified that hit compounds NPC216682 and NPC480509 exhibited higher whereas NPC210910 and NPC247082 showed moderate stabilization and predicted inhibition of SIRT1 protein.

NP216682 is a quinolizidine alkaloid from plant species including *Tephrosia tinctoria, Allanblackia stuhlmannii, Boehmeria caudata*, and *Chaenomeles japonica*. It has a structure consisting of two fused piperidine rings and an acetylated piperidine moiety and is thus made up of a fused nitrogen-rich, dense non-aromatic heterocycle. There has been no published research relating to any form of biological activity for this compound. NPC480509 (Praziquantel), a widely used anthelmintic agent derived from *Baccharis lateralis*, belongs to the tetrahydroisoquinoline family of compounds and has been shown to have synergistic anticancer properties with paclitaxel through the induction of mitotic arrest and apoptosis in neoplastic cells (Wu et al., 2012). NPC210910 is a quinazoline-containing compound isolated from a variety of sources, including *Necturus maculosus, Melolontha vulgaris, Licaria rigida*, and *Haplophyton cimicidum*. The structure consists of a core quinazolinone ring, an amino group substituted on a dihydropyrrole ring, and a benzoic acid substituent. The biological activity of this compound is unknown. NPC247082 is a bis-indole/tryptophan alkaloid linked by an isocaproic acid to 2 indole moieties isolated from the *Beilschmiedia zenkeri* plant. Like many of the other hits described above, there is no published activity data for this compound, emphasizing the exploratory nature of the study.

The preliminary experimental validation using MTT assay of the only available test compound (Praziquantel) among other hits, revealed the cytotoxic nature on MDA-MB-231 and MCF7 breast cancer cell lines and the non-cytotoxic nature on normal breast cell lines (MCF10A). The limitation of this study includes the utilization of external or decoy datasets to further understand the robustness of the models, SIRT1-based inhibition (enzymatic assays), other SIRT-isoforms inhibition to answer the selectivity of Praziquantel and other hits, gene-expression analysis, and Western blotting assay to confirm the inhibition of SIRT1 in breast cancer cells. The current evidence supports preliminary identification of potential candidates, but not definitive conclusions regarding therapeutic efficacy or mechanism of action. Moreover, Praziquantel, along with other hits and standard SIRT1 inhibitors as positive controls, may be evaluated *in-vitro* and *in vivo* for a complete mechanism of action, as part of this study’s future scope. When used in a pharmaceutical framework different from conventional techniques, the integrated *in silico* and *in-vitro* methodology suggested in this study could produce novel results.

## 5 Conclusion

Epigenetic modulators, SIRT1, deacetylate tumor suppressor genes such as p53, making them non-functional. SIRT1 protein is upregulated in TNBC cells. However, context-specific differences also exist. Several studies suggested that the inhibition of SIRT1 protein may favor normal functioning of such tumor suppressor genes and ultimately help in combating TNBC. This study explored advanced computational techniques, including ligand-based drug design (three-stage ML modelling, explanation of the developed ML models, applicability domain of the QDA + ROS and XGBClassifier + ROS ML models, and prediction of an unknown dataset) and structure-based drug design (primary level virtual screening, secondary level virtual screening, and molecular dynamic simulation studies). These rigorous evaluations helped us to identify four natural compounds with high stability (NPC216682 and NPC480509) and moderate stability (NPC210910 and NPC247082) upon binding with the SIRT1 protein. These hit compounds may effectively inhibit the SIRT1 proteins compared to the existing options. Among the four hit compounds, three compounds (NPC216682, NPC210910, and NPC247082) have not been reported for any activity so far. In contrast, the hit NPC480509 (Praziquantel) has been reported for its anthelmintic and anticancer properties. The MTT assay confirmed the cytotoxic nature of Praziquantel on MDA-MB-231 and MCF7 breast cancer cell lines and the non-cytotoxic nature on normal breast cell lines (MCF10A) as the preliminary step. The Praziquantel test compound, along with other predicted SIRT1 protein inhibitors, may be further evaluated for SIRT1 inhibition in TNBC cell lines by *in-vitro* and *in vivo* experiments to answer the selectivity of SIRT1 protein. Therefore, this study predicts four natural compounds that can be used for targeting SIRT1 inhibition and ultimately managing TNBC.

## Data Availability

All the codes used for building machine learning models have been deposited to out GitHub repository: https://github.com/deepsharma26/SIRT1_ML_NPASS.
