# Supplementary material for: An integrative machine learning, explainable AI, molecular simulation, and cytotoxicity validation framework for the discovery of selective SIRT1 inhibitors against triple negative breast cancer
Source: Front Bioinform. 2026 Jun 10;6:1827014. doi: 10.3389/fbinf.2026.1827014 (PMC13291546; doi:10.3389/fbinf.2026.1827014)
Supplement: Supplementary file 1 [file Table1.docx]

**An Integrative Machine Learning, Explainable AI, Molecular Simulation, and Cytotoxicity Validation Framework for the Discovery of Selective SIRT1 Inhibitors against Triple Negative Breast Cancer**

**Table S1** Top 20 features along with their MI core

| **S. no.** | **Features** | **MI Scores** | **S. no.** | **Features** | **MI Scores** |
| --- | --- | --- | --- | --- | --- |
| 1 | MACCSFP118 | 0.0487 | 11 | MACCSFP73 | 0.0255 |
| 2 | MACCSFP82 | 0.0436 | 12 | MACCSFP108 | 0.0232 |
| 3 | MACCSFP138 | 0.0428 | 13 | MACCSFP54 | 0.0214 |
| 4 | MACCSFP100 | 0.0378 | 14 | MACCSFP52 | 0.0209 |
| 5 | MACCSFP125 | 0.0322 | 15 | MACCSFP113 | 0.0204 |
| 6 | MACCSFP137 | 0.0317 | 16 | MACCSFP92 | 0.0198 |
| 7 | MACCSFP140 | 0.0304 | 17 | MACCSFP79 | 0.0197 |
| 8 | MACCSFP90 | 0.0294 | 18 | MACCSFP129 | 0.0195 |
| 9 | MACCSFP152 | 0.0287 | 19 | MACCSFP91 | 0.0185 |
| 10 | MACCSFP97 | 0.0279 | 20 | MACCSFP128 | 0.0182 |

**Table S2** Stage 1 ML modeling using the Lazy Classifier Python module

| **Model** | **Accuracy** | **Balanced Accuracy** | **ROC AUC** | **F1 Score** | **Time Taken** |
| --- | --- | --- | --- | --- | --- |
| Label Spreading | 0.84375 | 0.735366823 | 0.735366823 | 0.846876787 | 0.049524546 |
| Label Propagation | 0.84375 | 0.735366823 | 0.735366823 | 0.846876787 | 0.048429728 |
| Random Forest Classifier | 0.857638889 | 0.72653836 | 0.72653836 | 0.855732569 | 0.208427906 |
| LGBM Classifier | 0.850694444 | 0.722388982 | 0.722388982 | 0.850046287 | 0.107499123 |
| Bagging Classifier | 0.850694444 | 0.722388982 | 0.722388982 | 0.850046287 | 0.040251493 |
| Bernoulli NB | 0.760416667 | 0.719828728 | 0.719828728 | 0.784290748 | 0.012564659 |
| Quadratic Discriminant Analysis | 0.788194444 | 0.71929902 | 0.71929902 | 0.804649559 | 0.0165627 |
| Decision Tree Classifier | 0.840277778 | 0.716164916 | 0.716164916 | 0.841608796 | 0.017272949 |
| Extra Trees Classifier | 0.854166667 | 0.715900062 | 0.715900062 | 0.851526063 | 0.158466339 |
| XGB Classifier | 0.847222222 | 0.711750684 | 0.711750684 | 0.845877251 | 0.333408117 |
| Gaussian NB | 0.732638889 | 0.703231217 | 0.703231217 | 0.762151034 | 0.016736269 |
| Perceptron | 0.788194444 | 0.702171802 | 0.702171802 | 0.802709008 | 0.016410351 |
| K Neighbors Classifier | 0.795138889 | 0.69775757 | 0.69775757 | 0.807081678 | 0.016857147 |
| Nearest Centroid | 0.694444444 | 0.697536859 | 0.697536859 | 0.732246295 | 0.016679049 |
| Linear Discriminant Analysis | 0.850694444 | 0.696698155 | 0.696698155 | 0.845756548 | 0.046364784 |
| Extra Tree Classifier | 0.826388889 | 0.690738942 | 0.690738942 | 0.827835648 | 0.016884327 |
| Passive Aggressive Classifier | 0.788194444 | 0.642226538 | 0.642226538 | 0.793973568 | 0.023490191 |
| SVC | 0.864583333 | 0.636488037 | 0.636488037 | 0.840889487 | 0.044283628 |
| Logistic Regression | 0.847222222 | 0.608987375 | 0.608987375 | 0.821924373 | 0.023756027 |
| Ada Boost Classifier | 0.850694444 | 0.602498455 | 0.602498455 | 0.821624475 | 0.138318062 |
| Linear SVC | 0.850694444 | 0.585371237 | 0.585371237 | 0.815092092 | 0.017799854 |
| Calibrated Classifier CV | 0.850694444 | 0.55968041 | 0.55968041 | 0.80334705 | 0.085424662 |
| Ridge Classifier CV | 0.850694444 | 0.55968041 | 0.55968041 | 0.80334705 | 0.017513514 |
| Ridge Classifier | 0.847222222 | 0.557605721 | 0.557605721 | 0.80096917 | 0.017640829 |
| Dummy Classifier | 0.836805556 | 0.5 | 0.5 | 0.762457992 | 0.015224695 |
| SGD Classifier | 0.680555556 | 0.458020659 | 0.458020659 | 0.692529516 | 0.024124861 |

**Table S3** Stage 2 ML modeling with top 10 models

| **Model** | **Accuracy** | **BA** | **ROC AUC** | **Cohen's Kappa** | **MAE** | **Precision (0)** | **Precision (1)** | **Recall (0)** | **Recall (1)** | **F1 (0)** | **F1 (1)** |
| --- | --- | --- | --- | --- | --- | --- | --- | --- | --- | --- | --- |
| Label Spreading | 0.8368 | 0.7055 | - | 0.4075 | 0.1631 | 0.90 | 0.50 | 0.90 | 0.51 | 0.90 | 0.51 |
| Label Propagation | 0.8368 | 0.7055 | 0.8604 | 0.4075 | 0.1631 | 0.90 | 0.50 | 0.90 | 0.51 | 0.90 | 0.51 |
| Random Forest Classifier | 0.8472 | 0.7031 | 0.8649 | 0.4207 | 0.1527 | 0.90 | 0.53 | 0.92 | 0.49 | 0.91 | 0.51 |
| LGBM Classifier | 0.8506 | 0.6795 | 0.8633 | 0.3964 | 0.1493 | 0.89 | 0.56 | 0.93 | 0.43 | 0.91 | 0.48 |
| Bagging Classifier | 0.8506 | 0.7223 | 0.8639 | 0.4486 | 0.1493 | 0.91 | 0.54 | 0.91 | 0.53 | 0.91 | 0.54 |
| Bernoulli NB | 0.7638 | 0.7304 | 0.7794 | 0.3477 | 0.2361 | 0.93 | 0.38 | 0.78 | 0.68 | 0.85 | 0.48 |
| QDA | 0.8159 | 0.7530 | 0.8414 | 0.4289 | 0.1840 | 0.93 | 0.46 | 0.85 | 0.66 | 0.89 | 0.54 |
| Decision Tree Classifier | 0.8368 | 0.6969 | 0.7926 | 0.3973 | 0.1631 | 0.90 | 0.50 | 0.90 | 0.49 | 0.90 | 0.49 |
| Extra Trees Classifier | 0.8506 | 0.7052 | 0.8396 | 0.4288 | 0.1493 | 0.90 | 0.55 | 0.92 | 0.49 | 0.91 | 0.52 |
| XGB Classifier | 0.8437 | 0.7280 | 0.8248 | 0.4597 | 0.1562 | 0.90 | 0.56 | 0.91 | 0.55 | 0.91 | 0.55 |

ODA: Quadratic Discriminant Analysis, BA: Balanced Accuracy, ROC-AUC: Receiver Operating Characteristic curve-Area Under the Curve, MAE: Mean Absolute Error

**Table S4** Stage 3 ML modeling with top 4 models, along with sampling strategies

| **Model** | **Accuracy** | **BA** | **ROC AUC** | **Cohen's Kappa** | **MAE** | **Precision (0)** | **Precision (1)** | **Recall (0)** | **Recall (1)** | **F1 (0)** | **F1 (1)** | **CV accuracy** | **Y-Randomization** | |
| --- | --- | --- | --- | --- | --- | --- | --- | --- | --- | --- | --- | --- | --- | --- |
|  |  |  |  |  |  |  |  |  |  |  |  |  | **Baseline accuracy** | **Avg. accuracy on RL** |
| RF Classifier + ROS | 0.7951 | 0.7919 | 0.8554 | 0.4377 | 0.2048 | 0.95 | 0.43 | 0.80 | 0.79 | 0.87 | 0.56 | 0.8576 ± 0.0390 | 0.8852 | 0.6775 |
| RF Classifier + Undersampling | 0.7013 | 0.7701 | 0.8507 | 0.3307 | 0.2986 | 0.96 | 0.34 | 0.67 | 0.87 | 0.79 | 0.49 | 0.7273 ± 0.0823 | 0.9120 | 0.8239 |
| Bagging Classifier + ROS | 0.7951 | 0.7833 | 0.8534 | 0.4304 | 0.2048 | 0.95 | 0.43 | 0.80 | 0.77 | 0.87 | 0.55 | 0.8478 ± 0.0416 | 0.8852 | 0.6753 |
| Bagging Classifier + Undersampling | 0.7152 | 0.7699 | 0.8578 | 0.3411 | 0.2847 | 0.96 | 0.35 | 0.69 | 0.85 | 0.80 | 0.49 | 0.7459 ± 0.0676 | 0.9120 | 0.8268 |
| QDA + ROS | 0.8195 | 0.8195 | 0.9091 | 0.6390 | 0.1804 | 0.79 | 0.86 | 0.88 | 0.76 | 0.83 | 0.81 | 0.7318 ± 0.0644 | 0.9120 | 0.8272 |
| QDA + Undersampling | 0.7534 | 0.7499 | 0.8295 | 0.3564 | 0.2465 | 0.94 | 0.37 | 0.76 | 0.74 | 0.84 | 0.50 | 0.7318 ± 0.0644 | 0.8611 | 0.7354 |
| XGB Classifier + ROS | 0.8651 | 0.8651 | 0.9332 | 0.7302 | 0.1348 | 0.85 | 0.88 | 0.88 | 0.85 | 0.87 | 0.86 | 0.7273 ± 0.0686 | 0.8611 | 0.7331 |
| XGB Classifier + Undersampling | 0.7430 | 0.7950 | 0.8412 | 0.3855 | 0.2569 | 0.97 | 0.38 | 0.72 | 0.87 | 0.82 | 0.53 | 0.7273 ± 0.0686 | 0.9074 | 0.8233 |

RF Classifier: Random Forest Classifier, QDA: Quadratic Discriminant Analysis, CV accuracy: Stratified 10-Fold CV Accuracies, Baseline (Real Labels) Accuracy, Avg. accuracy on RL: Average Accuracy on Randomized Labels

**Table S5** ADMET profile of the selected compounds

| Compounds | Formula | MW | HBA | HBD | GI absorption | Lipinski violations | PAINS alerts | Toxicity Class | Hepatotoxicity | Carcinogenicity | Immunotoxicity | Mutagenicity | Cytotoxicity |
| --- | --- | --- | --- | --- | --- | --- | --- | --- | --- | --- | --- | --- | --- |
| NPC113333 | C24H21N3 | 351.44 | 0 | 3 | High | 0 | 0 | 3 | Inactive (0.66) | Active (0.69) | Inactive (0.98) | Active (0.86) | Inactive (0.79) |
| NPC216682 | C22H35N3O | 357.53 | 3 | 0 | High | 0 | 0 | 4 | Inactive (0.9) | Inactive (0.66) | Inactive (0.99) | Inactive (0.66) | Inactive (0.80) |
| NPC479631 | C26H28N2O4 | 432.51 | 5 | 0 | High | 0 | 0 | 4 | Active (0.69) | Inactive (0.62) | Active (0.96) | Inactive (0.97) | Inactive (0.93) |
| NPC280116 | C22H33NO3 | 359.5 | 4 | 2 | High | 0 | 0 | 4 | Inactive (0.86) | Active (0.51) | Active (0.95) | Inactive (0.65) | Inactive (0.86) |
| NPC154993 | C21H20N4OS | 376.47 | 4 | 2 | High | 0 | 0 | 4 | Inactive (0.63) | Inactive (0.6) | Active (0.98) | Inactive (0.61) | Inactive (0.58) |
| NPC480509 | C19H24N2O2 | 312.41 | 2 | 0 | High | 0 | 0 | 5 | Inactive (0.95) | Inactive (0.84) | Inactive (0.97) | Inactive (0.9) | Inactive (0.64) |
| NPC22587 | C21H20N4O2S | 392.47 | 4 | 2 | High | 0 | 0 | 4 | Inactive (0.68) | Active (0.51) | Active (0.95) | Inactive (0.5) | Inactive (0.6) |
| NPC71848 | C26H28N2O5 | 448.51 | 6 | 1 | High | 0 | 0 | 3 | Inactive (0.77) | Inactive (0.56) | Active (0.99) | Inactive (0.63) | Inactive (0.52) |
| NPC210910 | C19H16N4O3 | 348.36 | 5 | 3 | High | 0 | 0 | 4 | Inactive (0.57) | Inactive (0.56) | Inactive (0.94) | Inactive (0.5) | Inactive (0.7) |
| NPC83511 | C19H13NO5 | 335.31 | 5 | 0 | High | 0 | 0 | 4 | Inactive (0.78) | Active (0.61) | Active (0.96) | Active (0.57) | Active (0.6) |
| NPC247082 | C22H22N2O2 | 346.42 | 2 | 3 | High | 0 | 0 | 4 | Inactive (0.57) | Inactive (0.68) | Inactive (0.99) | Inactive (0.71) | Inactive (0.79) |

MW: Molecular weight, HBA: H-bond acceptors, HBD: H-bond donors

**Table S6** MTT assay for the test compound Praziquantel (NPC480509) on MDA-MD-231, MCF7, and MCF10A cell lines

| **Cell lines** | **Conc** | **12.5 µg/ml** | **25 µg/ml** | **50 µg/ml** | **100 µg/ml** | **200 µg/ml** | **Control** |
| --- | --- | --- | --- | --- | --- | --- | --- |
| **MDA-MB-231** | ABS | 0.472 | 0.391 | 0.304 | 0.221 | 0.132 | 0.565 |
|  |  | 0.474 | 0.392 | 0.305 | 0.224 | 0.134 | 0.568 |
|  |  | 0.476 | 0.394 | 0.302 | 0.225 | 0.131 | 0.564 |
|  | **Avg** | **0.474** | **0.392** | **0.304** | **0.223** | **0.132** | **0.566** |
| **MCF7** | ABS | 0.435 | 0.324 | 0.233 | 0.118 | 0.055 | 0.526 |
|  |  | 0.432 | 0.325 | 0.234 | 0.117 | 0.057 | 0.524 |
|  |  | 0.436 | 0.326 | 0.237 | 0.119 | 0.058 | 0.521 |
|  | **Avg** | **0.434** | **0.325** | **0.235** | **0.118** | **0.057** | **0.524** |
| **MCF10A** | ABS | 0.418 | 0.411 | 0.403 | 0.398 | 0.392 | 0.422 |
|  |  | 0.415 | 0.412 | 0.405 | 0.399 | 0.391 | 0.423 |
|  |  | 0.416 | 0.412 | 0.402 | 0.396 | 0.392 | 0.421 |
|  | **Avg** | **0.416** | **0.412** | **0.403** | **0.398** | **0.392** | **0.422** |

**Table S7** Percentage cell inhibition for the test compound Praziquantel (NPC480509) on MDA-MD-231, MCF7, and MCF10A cell lines

| **Conc (µg/ml)** | **% Cell inhibition (MDA-MB-231)** | **% Cell inhibition (MCF7)** | **% Cell Growth (MCF10A)** |
| --- | --- | --- | --- |
| 12.5 | 16.1 | 17.01 | 98.65719 |
| 25 | 30.61 | 37.85 | 97.55134 |
| 50 | 46.37 | 55.25 | 95.57662 |
| 100 | 60.53 | 77.43 | 94.23381 |
| 200 | 76.63 | 89.29 | 92.81201 |
